# Improving cognitive impairment through chronic consumption of natural compounds/extracts: a systematic review and meta-analysis of randomized controlled trials

**DOI:** 10.3389/fnagi.2024.1531278

**Published:** 2025-01-30

**Authors:** Long Ngo Hoang, Haesung Lee, Sook Jeong Lee

**Affiliations:** Department of Bioactive Material Sciences and Research Centre of Bioactive Materials, Jeonbuk National University, Jeonju, Jeonbuk-do, Republic of Korea

**Keywords:** aging, Alzheimer’s disease, cognitive dysfunction, meta-analysis, neurodegeneration

## Abstract

**Introduction:**

This systematic review and meta-analysis aimed to compare the efficacy of extended supplementation (≥6 weeks) with natural compounds or extracts in improving cognitive function in patients with mild cognitive impairment (MCI) or Alzheimer’s disease (AD).

**Methods:**

A comprehensive literature search was conducted across Cochrane, PubMed, PsycARTICLES, Scopus, and Web of Science databases from inception to April 10, 2024. Eligible studies were randomized controlled trials evaluating cognitive outcomes in patients with MCI or AD using the Mini-Mental State Examination (MMSE) and the Alzheimer’s Disease Assessment Scale-Cognitive Subscale (ADAS-Cog).

**Results:**

From an initial pool of 6,687 articles, 45 were deemed relevant for qualitative analysis. Of these, 37 studies demonstrated improvements or positive trends in cognitive outcomes with natural compound or extract supplementation. A total of 35 studies met the criteria for meta-analysis. The meta-analysis, involving 4,974 participants, revealed significant improvements in ADAS-Cog scores (pooled standardized mean difference = −2.88, 95% confidence interval [CI]: −4.26 to −1.50; t_24_ = −4.31, *p* < 0.01) following supplementation. Additionally, a suggestive trend toward improvement in MMSE scores was observed in a subgroup analysis of 1,717 participants (pooled standardized mean difference = 0.76, 95% CI: 0.06 to 1.46, t_18_ = 2.27, *p =* 0.04).

**Conclusion:**

These findings support the potential cognitive benefits of extended (≥6 weeks) supplementation with natural compounds or extracts in individuals with MCI or AD. Further research is warranted to confirm these results and elucidate the underlying mechanisms.

**Systematic review registration:**

https://www.crd.york.ac.uk/PROSPERO/.

## 1 Introduction

Alzheimer’s disease (AD) is a progressive neurodegenerative disorder that significantly affects individuals worldwide. It is characterized by a gradual decline in cognitive abilities, manifesting as memory loss, personality changes, and difficulties with daily functioning (Katzman, 1993). Mild cognitive impairment (MCI), often considered a precursor to AD, represents a stage of cognitive decline that does not yet meet the diagnostic criteria for dementia (Morris, 1997). With the global population aging, the prevalence of both AD and MCI is projected to rise significantly (Deary et al., 2009), posing critical challenges to healthcare systems and society at large.

The current treatment options for AD and MCI remain limited (Long and Holtzman, 2019), driving growing interest in exploring natural compounds and extracts as potential therapeutic interventions (Andrade et al., 2019). Natural compounds derived from plants, fruits, and vegetables have demonstrated promising properties, including anti-inflammatory, anti-oxidant, and neuroprotective effects (Wang et al., 2022). Recent studies have focused on elucidating the mechanisms through which these compounds and extracts may enhance cognitive function and provide neuroprotection against degenerative processes (Andrade et al., 2019).

Examples of natural compounds extensively studied for their neuroprotective effects include alkaloids, polyphenols, and terpenoids (Jiang et al., 2017). Flavonoids such as quercetin (Dastmalchi et al., 2008; Khan et al., 2019) and catechins (Ide et al., 2018) exhibit anti-inflammatory and anti-oxidant properties that safeguard neurons from oxidative stress and inflammation. Polyphenols, including resveratrol (Lee et al., 2017; Sawda et al., 2017; Turner et al., 2015) and curcumin (Hamaguchi et al., 2010; Ono et al., 2004; Rainey-Smith et al., 2016), have shown potential in improving cognitive function and protecting against neurodegeneration. Similarly, terpenoids such as ginsenosides (Heo et al., 2012; Heo et al., 2016; Lee et al., 2008; Lee et al., 2022; Park H. et al., 2019; Sheng et al., 2015) have been reported to improve memory and cognitive function through neuroprotective mechanisms.

Randomized controlled trials (RCTs) have assessed the efficacy of natural compounds and extracts in improving cognitive function and slowing the progression of AD and MCI (Akhondzadeh et al., 2003a, 2003b; Akhondzadeh et al., 2010a; Akhondzadeh et al., 2010b; Heo et al., 2012; Lee et al., 2008; Muangpaisan et al., 2022; Noguchi-Shinohara et al., 2020; Tsolaki et al., 2016; Wang et al., 2018). Among the most extensively investigated natural extracts are *Ginkgo biloba* (DeKosky et al., 2008; Gauthier and Schlaefke, 2014; Herrschaft et al., 2012; Hofferberth, 1994; Ihl et al., 2011; Kanowski and Hoerr, 2003; Le Bars et al., 1997; Le Bars et al., 2000; Le Bars et al., 2002; Li et al., 2023; Lopez et al., 2019; Maurer et al., 1997; Mazza et al., 2006; Schneider et al., 2005; Shi et al., 2010; Snitz et al., 2009) and *Curcuma longa* (Baum et al., 2008; Obulesu and Rao, 2011; Ono et al., 2004; Rainey-Smith et al., 2016). Both have demonstrated potential in enhancing global cognitive function and protecting against cognitive decline.

This systematic review and meta-analysis aim to synthesize the current evidence from RCTs on the effects of natural compounds and extracts on cognitive function in individuals with AD or MCI. By evaluating their therapeutic potential, this study seeks to provide a comprehensive overview of the current state of knowledge and assess the feasibility of incorporating these natural agents into treatment strategies for AD and MCI.

## 2 Methods

### 2.1 Inclusion criteria

This study applied the population, intervention, comparator, outcome, and study design framework (PICOS) (Supplementary Table S1) to establish the inclusion criteria for relevant studies. Eligible studies met the following criteria: (1) study design: Randomized controlled trials including parallel or multi-arm trials; (2) participants: Patients diagnosed with AD or cognitive impairment according to established diagnostic criteria, such as the *Diagnostic and Statistical Manual of Mental Disorders (DSM)*, the *National Institute of Neurological and Communicative Disorders and Stroke and the Alzheimer’s Disease and Related Disorders Association (NINCDS-ADRDA)*, or the *International Classification of Diseases (ICD)*; (3) Intervention and control groups: An experimental group receiving natural compounds or extracts, compared with a control group receiving a placebo, equivalent, or standard treatment; (4) Outcome measures: Cognitive outcomes assessed via the Mini-Mental State Examination (MMSE) and/or the Alzheimer Disease Cooperative Study-Activities of Daily Living Scale (ADAS-cog). Exclusion criteria included studies that: (1) were derived from the same trial; (2) Lacked analyzable data; (3) Were not available in full-text format; (4) Were not published in English; (5) Combined multiple natural compounds or extracts in the intervention.

### 2.2 Data sources

The systematic review followed guidelines outlined in the *Cochrane Handbook for Systematic Reviews of Interventions* (version 6.3; Higgins et al., 2022) and the *Centre for Reviews and Dissemination* (University of York, 2009). The study adhered to the preferred reporting items for systematic reviews and meta-analysis framework (PRISMA) (Supplementary Table S2; Moher et al., 2009). The review protocol was registered in International prospective register of systematic reviews (PROSPERO) under registration number CRD42022369293. A systematic search was conducted across Cochrane, PubMed, PsycARTICLES, Scopus, and Web of Science databases from inception to April 10, 2024. The search strategy included the following terms: (Alzheimer’s disease OR Alzheimer dementia) AND (natural OR compound OR flower OR plant OR extract* OR powder OR oil) AND (cognition OR cognitive function OR cognit*).

### 2.3 Study selection

Two independent reviewers screened articles for eligibility using EndNote X9 for reference management. First, titles and abstract were screened for relevance. Full texts of potentially eligible studies were then reviewed. Reference lists from included studies and relevant systematic reviews were also hand-searched to identify additional eligible articles.

### 2.4 Risk of bias and quality assessment

The risk of bias was assessed using the Cochrane Risk of Bias tool (RoB2), which evaluates aspects such as randomization, deviations from intended interventions, missing outcome data, outcome measurement, and selective reporting (Sterne et al., 2019). Studies were categorized as having low risk, some concerns, or high risk of bias. When applicable, funnel plots and Egger’s test (Egger et al., 1997) were employed to evaluate potential publication bias.

### 2.5 Data collection

A standardized form was used to extract the following data: Publication information (authors, title, year); Study characteristics (design and number of participants); Participant characteristics (drug type, dosage, and duration of intervention); Cognitive outcomes (mean values and standard deviations for MMSE and ADAS-cog scores). When data were presented as means with 95% confidence intervals (CIs) or as medians with interquartile ranges, these were converted into means and standard deviations (SDs) using methods from the *Cochrane Handbook* (Chapter 6.5.2; Higgins et al., 2022) or Wan et al.’s formulas (Wan et al., 2014).

### 2.6 Data synthesis

Pooled data were analyzed using R (version 4.3.3) with the “meta” package. For long-term studies (≥ 6 weeks), endpoint and baseline data were used to calculate mean differences for intervention and control groups. Results were presented in forest plots as weighted mean differences or standardized mean differences (SMDs) with 95% CIs and two-sided *p* values. Subgroup analyses were conducted to explore variations in study designs and characteristics.

Global cognitive outcomes were evaluated using: (a) MMSE, scores range from 0 to 30, with higher scores indicating better cognition; and (b) ADAS-cog, scores range from 0 to 70, with higher scores indicating greater cognitive impairment. Effect sizes were calculated as Hedges’ g, classified as very small (< 0.2), small (0.2–0.5), moderate (0.5–0.8), and large (> 0.8; Hedges, 2009). The Hartung-Knapp-Sidik-Jonkman random-effects model was used to account for heterogeneity in treatment effects (Inthout et al., 2014).

Statistical heterogeneity was assessed using the chi-squared (*χ*^2^) test and *I^2^* statistic. *I^2^* value ≥50% indicated moderate heterogeneity, while values between 75 and 100% suggested substantial heterogeneity (Higgins et al., 2003). Sensitivity analyses were performed by systematically excluding studies to identify potential outliers influencing the overall effect size. Statistical significance was set at *p* < 0.05 for all analyses.

## 3 Results

### 3.1 Literature search

**Figure 1:**
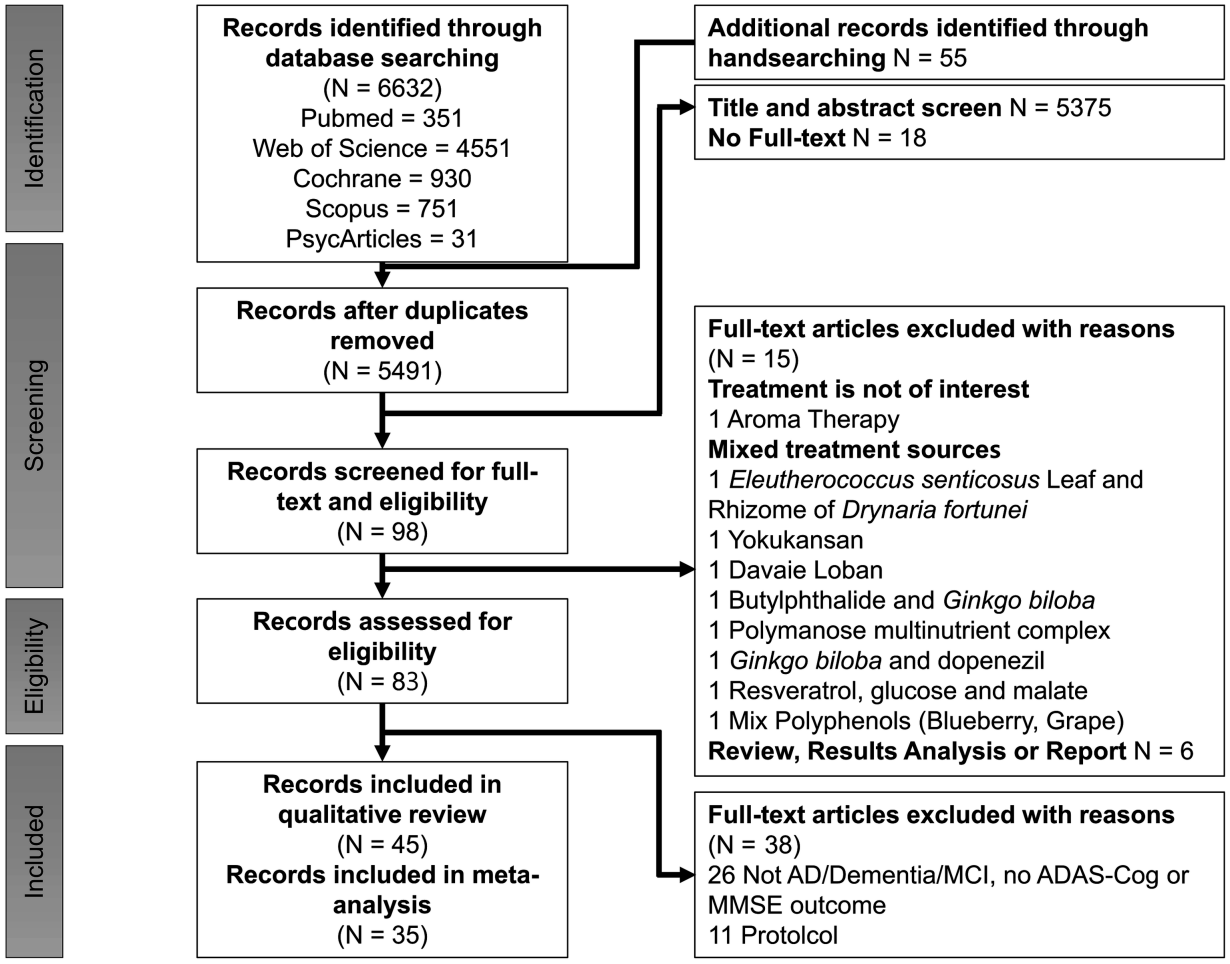
PRISMA flow diagram for study selection.

The study selection process is illustrated in Figure 1, adhering to PRISMA guidelines (Moher et al., 2009). A comprehensive search across five databases yielded 6,687 articles, supplemented by 55 additional articles identified through manual searches of reference lists from relevant studies. After the removal of duplicates, 5,491 articles remained. Of these, 5,393 were excluded for not being human intervention studies, randomized controlled trials or for only having abstract-level information available. Following the screening of titles and abstracts, 98 articles evaluating cognitive function were shortlisted for full-text review. Subsequently, 53 articles were excluded due to not meeting inclusion criteria. Ultimately, 45 trials were included in the qualitative review, of which 35 provided sufficient data for meta-analysis.

### 3.2 Qualitative analysis and study characteristics

The systematic review incorporated 45 studies, with a detailed summary provided in Table 1. These studies addressed various dimensions, including study quality, sample size, participant characteristics (e.g., health status, diagnostic criteria), intervention types, dosages, durations, cognitive assessment measures, and key outcome metrics.

**Table 1:** Summary of interventions assessing the effects of natural compounds/extracts on cognition.

References	Country	Study design	Sample (Number of subjects, mean age (years), male (%), health status)	Intervention type, dose, and duration	Cognitive tasks	Outcome
Wade et al. (2014)	UK/USA	RCT, double-blind	*N = 80;* 75; male 50%; Mild–Moderate AD^a^	Add-on prolonged-release melatonin 2 mg or placebo, 24 weeks	ADAS-cog, MMSE, IADL, PSQI, CGI, NPI, WHO-5, SDI	Safe and well tolerated; Improvement of IADL, MMSE, PSQI; No significant improvement of ADAS-Cog
Kaddoumi et al. (2022)	USA	RCT, double-blind	*N = 25;* 66; male 30%; MCI (WMS-IV)	Extra-virgin olive oil 30 mL/day, refined olive oil 30 mL/day, 26 weeks	MMSE, CDR	Improvement of CDR. Enhances brain connectivity and reduces BBB permeability.
Hofferberth (1994)	Germany	RCT, double-blind	*N = 40;* 63; male 50%; Dementia/AD^a^	*Ginkgo biloba* 80 mg or placebo daily, 12 weeks	SKT, SCAG, Saccade Test	Safe and well tolerated; Improvement of SKT and Saccade Test
van et al. (2000)	USA	RCT, double-blind	*N = 176;* 72; male 45%; Mild–Moderate AD: (NINCDS-ADRDA)	Physostigmine 24 or 30 mg/day or placebo, 12 weeks	MMSE, ADAS-cog, CGIC, IADL, CIBIC+	Improvement of ADAS-cog, and CIBIC+. No significant improvement of MMSE, CGIC, and IADL. Adverse events: nausea and vomiting 47.0% of all physostigmine-treated subjects
Boespflug et al. (2018)	USA	RCT, double-blind	*N = 16;* 78; male 54%; MCI (NINCDS-ADRDA)	25 g 50% *Vaccinium ashei* Reade, 50% *Vaccinium corymbosum* L. or placebo, 16 weeks	MoCA, VLT, GAS, GAI	Improvement of blood oxygen level-dependence; no clear indication of working memory enhancement
Calapai et al. (2017)	Italy	RCT, double-blind	*N = 111;* 66; male 48%; MCI^a^	*Vitis vinifera* powder 250 mg/day or placebo, 12 weeks	MMSE, RBANS	Improvement of MMSE and RBANS
Wilcock et al. (2000)	Europe/Canada	RCT, double-blind	*N = 653;* 72; male 37%; Mild–Moderate AD (NINCDS-ADRDA)	Galantamine 24, 32 mg or placebo daily, 26 weeks	ADAS-cog	Improvement of ADAS-cog
Herrschaft et al. (2012)	Germany	RCT, double-blind	*N = 410;* 65; male 30%; Mild–Moderate AD with neuropsychiatric (NINCDS-ADRDA)	EGb 240 mg or placebo daily, 24 weeks	ADCS-ADL CGIC, SKT, NPI, DEMQOL-Proxy, VFT	Safe; Improvement of SKT and NPI
Quinn et al. (2010)	USA	RCT, double-blind	*N = 402*; 76; male 48%; Mild–Moderate AD^b^	Algal DHA 2 g/d or placebo, 78 weeks	ADAS-cog, MMSE, CDR, NPI, ADCS-ADL	No improvement of ADAS-cog and CDR
Lee et al. (2017)	USA	RCT, double-blind	*N = 10;* 72; male 50%; MCI^a^	*Vitis vinifera* 72 g/day or placebo, 26 weeks	ADAS-cog, MMSE, VLT, WCST, WAIS-III, WATR, CFT	No improvement in cognitive measures; Maintains cerebral metabolism; Delays decline in left prefrontal, cingulate, and left superior posterolateral temporal cortex
Maurer et al. (1997)	Germany	RCT, double-blind	*N = 20*; 68; male 50%; Mild–Moderate AD (DSM-III-R)	EGb 240 mg/day or placebo, 12 weeks	SKT, ADAS-cog, ADAS-noncog, CGI (item 2)	Improvement of cognitive functions
Rockwood et al. (2001)	UK/USA/Canada	RCT, double-blind	*N = 386;* 75; male 64%; Mild–Moderate AD (NINCDS-ADRDA)	Dose escalation of galantamine from 8 to 24–32 mg (individual case) or placebo daily, 12 weeks	ADAS-cog, CIBIC+, ADL	Improvement in cognitive measures
Park H. et al. (2019)	South Korea	RCT, double-blind	*N = 90;* 61; male 33.3%; MCI (Petersen criteria)	*Panax ginseng* powder 3 g/day or placebo, 24 weeks	MMSE, IADL, LVT, RCFT	Safe; Improvement of RCFT and RCFT 20-min delayed recall
Shin et al. (2009)	South Korea	RCT, double-blind	*N = 58;* 67; male 18%; MCI^a^	*Polygala tenuifolia* Willdenow extract (BT-11) 300 mg or placebo daily, 8 weeks	MMSE, CERAD	Improvement of CERAD
Lee et al. (2020)	South Korea	RCT, double-blind	*N = 53;* 60; male 23%; Subjective/MCI^a^	PhytoMeal (desalted *Salicornia europaea L.*)-ethanol extract 600 mg or placebo daily, 12 weeks	ADAS-cog	Safe; Improvement of frontal executive function in the patients with MCI.
Thal et al. (1999)	USA	RCT, double-blind	*N = 475;* 72; male 45%; Mild–Moderate AD (NINCDS-ADRDA)	Controlled release physostigmine 30 or 36 mg or placebo daily, 24 weeks	ADAS-cog, CIBIC, CGIC	Improvement of ADAS-cog and CIBIC+; No significant difference on CGIC; Adverse events: nausea, vomiting, diarrhea, anorexia, dyspepsia, and abdominal pain
Lee et al. (2013)	Malaysia	RCT, double-blind	*N = 36;* 66; male 20%; MCI^a^	DHA 1.3 g or 0.45 g eicosapentaenoic acid (EPA) placebo, 52 weeks	MMSE, CDT, GDS, RAVLT, VR, WMS-R	Safe and well tolerated; Improvement in short-term, working memory, immediate verbal memory, and delayed recall capability.
Rasi Marzabadi et al. (2022)	Iran	RCT, double-blind	*N = 60;* 75; male 22%; Mild–Moderate AD (NINCDS-ADRDA)	*Crocus sativus* L. 30 mg/day or donepezil 30 mg/day, 12 weeks	MMSE	No significant difference between two groups; Reduce inflammation and oxidative stress in treatment group
Schneider et al. (2005)	USA	RCT, double-blind	*N = 410;* 68; male 46%; Dementia (NINCDS-ADRDA)	*Ginkgo biloba* extract 120 mg or 240 mg, or placebo daily, 26 weeks.	ADAS-cog	Improvement in subgroup of patients with neuropsychiatric symptoms
Farokhnia et al. (2014)	Iran	RCT, double-blind	*N = 64;* 77; male 55%; Moderate–Severe AD (DSM-IV)	*Crocus sativus* L. 30 mg/day or memantine 20 mg/day, 52 weeks	MMSE, SCIRS, FAST	No significant difference between two groups
Raskind et al. (2000)	USA	RCT, double-blind	*N = 636;* 75; male 38%; Mild–Moderate AD (NINCDS-ADRDA)	Galantamine from 24, 32 mg or placebo daily, 26 weeks	ADAS-cog, CIBIC+	Improvement in cognitive measures
Mazza et al. (2006)	Italy	RCT, double-blind	*N = 76;* 68; male 46%; Mild–Moderate dementia (DSM-IV)	*Ginkgo biloba* 160 mg/day, donepezil 5 mg/day or placebo, 24 weeks	MMSE, SKT, CGI (item 2)	Improved cognitive function; No differences in the efficacy of EGb 761 and donepezil
Farlow et al. (2019)	USA	RCT, double-blind	*N = 141;* 71; male 49%; Moderate–Severe AD^a^	7 intravenous infusion (45 ± 5min) doses of Bryostatin 24 μg, 48 μg, first 2 doses (week 0, and 1), and 20 μg, 40 μg last 5 doses (week 3, 5, 7, 9, and 11), or placebo, 12 weeks	SIB	Safe; Improvement of SIB
Rafii et al. (2011)	USA	RCT, double-blind	*N = 210;* 72; male 45%; Mild–Moderate AD (NINCDS-ADRDA)	Huperzine A 200 μg or 400 μg or placebo daily, 16 weeks	ADAS-cog, MMSE, NPI	No improvement of ADAS-cog (200 μg); Improvement of ADAS-cog (400 μg)
Noguchi-Shinohara et al. (2020)	Japan	RCT, double-blind	*N = 23;* 72; male 52.17%; Mild AD (NIA-AA)	*Melissa officinalis* one capsule (500 mg rosmarinic acid) or placebo daily, 24 weeks	ADAS-cog, MMSE, CDR, DAD, NPI-Q	No improvement in cognitive measures; Improvement of NPI-Q
Noguchi-Shinohara et al. (2023)	Japan	RCT, double-blind	*N = 323;* 71; male 45%; Subjective/MCI (DSM-V)	*Melissa officinalis* one capsule (500 mg rosmarinic acid) or placebo daily, 96 weeks	ADAS-cog, MMSE, CDR-SB	No improvement in cognitive measures; May help prevent cognitive decline in older adults without hypertension
Le Bars et al. (1997)	USA	RCT, double-blind	*N* = 327; 68; male 46%; AD and multi-infarct dementia (DSM-III-R)	EGb 120 mg/day or placebo, 52 weeks	ADAS-cog, GERRI, CGIC	Improvement of ADAS-cog and GERRI
Le Bars et al. (2002)	USA	RCT, double-blind	*N = 236;* 68; male 42%; AD and multi-infarct dementia (DSM-III-R)	EGb 120 mg/day or placebo, 52 weeks	ADAS-cog, GERRI	Improvement of ADAS-cog and GERRI
Tariot et al. (2000)	USA	RCT, double-blind	*N = 978;* 77; male 64%; Mild/Moderate AD (NINCDS-ADRDA)	Galantamine of 8, 16, 24 mg or placebo daily, 22 weeks	ADAS-cog, CIBIC+	Improvement in cognitive measures
Ihl et al. (2011)	Germany	RCT, double-blind	*N = 410;* 65; male 32%; Mild–Moderate AD with neuropsychiatric (NINCDS-ADRDA)	EGb 240 mg or placebo daily, 24 weeks	ADCS-ADL CGIC, SKT, NPI, DEMQOL-Proxy, Verbal Fluency Test	Improvement of SKT and NPI
Turner et al. (2015)	USA	RCT, double-blind	*N = 119;* 71; male 54%; Mild–Moderate AD (NINCDS-ADRDA)	500 mg (QAM), 1,000 mg (500 mg BID), 1,500 mg (1,000 mg QAM, 500 mg QPM), 2000 mg (1,000 mg BID) Resveratrol dose escalation every 13 weeks or placebo, 52 weeks.	MMSE, CDR, ADAS-cog, NPI	Safe and well tolerated; No significant difference in cognitive measures between groups
Akhondzadeh et al. (2003a)	Iran	RCT, double-blind	*N = 35;* 73; male 57% Mild–Moderate AD (NINCDS-ADRDA) criteria	*Melissa officinalis* (at least 500 mg citral/ml) extract 60 drops/day or placebo 60 drops/day; 16 weeks	ADAS-cog, CDR-SB	Safe; Improvement of ADAS-cog, CDR-SB
Akhondzadeh et al. (2003b)	Iran	RCT, double-blind,	*N = 30;* 72; male 61%; Mild–Moderate AD (NINCDS-ADRDA)	*Salvia officinalis* extract 60 drops / day or placebo drop 60 drops / day, 16 weeks	ADAS-cog, CDR-SB	Safe; Improvement of ADAS-cog, and CDR-SB
Akhondzadeh et al. (2010a)	Iran	RCT, double-blind	*N = 44;* 72; male 54%; Mild–Moderate AD (DSM-IV and NINCDS-ADRDA)	Capsule *Crocus sativus L.* (Saffron) 30 mg / day (15 mg twice per day) or capsule of placebo (two capsules per day); 16 weeks	ADAS-Cog, CDR-SB	Safe; Improvement of ADAS-cog, and CDR-SD
Akhondzadeh et al. (2010b)	Iran	RCT, double-blind	*N = 54;* 73; Mild–Moderate AD (DSM-IV and NINCDS-ADRDA)	*Crocus sativus* 30 mg or donepezil 10 mg daily, 22 weeks	ADAS-cog, CDR-SB	No significant difference in cognitive measures between groups; Adverse event: vomiting in donepezil group
Erkinjuntti et al. (2002)	Finland	RCT, double-blind	*N = 592;* 75; male 53%; Mild–Moderate vascular Dementia (NINCDS-ADRDA and NINDS-AIREN)	Galantamine 24 mg or placebo daily, 26 weeks	ADAS-cog, CIBIC+	Improvement in cognitive measures
Tsolaki et al. (2016)	Greece	RCT, single-blind	*N = 35;* 70; male 25%; amnesic and multi domain MCI (Petersen and Winblad criteria)	*Crocus sativus*, 52 weeks^c^	GDS, FRSSD, NPI, MoCA, MMSE	Improvement of MMSE
Tsolaki et al. (2020)	Greece	RCT, double-blind	*N = 50;* 69; male 30%; MCI (Petersen criteria)	Greek High Phenolic Early Harvest Extra Virgin Olive Oil 50 mL/day, Moderate Phenolic 50 mL/day, Mediterranean Diet, 52 weeks	MMSE, ADAS-cog	Improvement in cognitive function
Muangpaisan et al. (2022)	Thailand	RCT, double-blind	*N = 102;* 77; male 31%; AD (DSM-IV-TR and NINCDS-ADRDA)	Mangosteen pericarp 4 to 8 mg/kg, 220 mg (≤ 55 kg), 24 weeks; 280 mg (> 55 kg), first 12 weeks, and 560 mg, last 12 weeks, or placebo daily, 24 weeks	ADAS-cog, ADCS-ADL, NPI-Q, CDR-SB	Safe and well tolerated; Improvement of ADAS-cog (low-dose); Reduced oxidative stress
Wang et al. (2018)	China	RCT^d^	*N = 42;* 75; male 30%; AD (NINCDS-ADRDA)	Spore Powder of *Ganoderma Lucidum* (SPGL); 4 capsules of 1,000 mg (250 mg/capsule) or placebo each time, 3 times daily, and 7 days weekly for a total of 6 weeks	ADAS-cog, WHOQOL-BREF, NPI	Safe and well tolerated; No improvement in cognitive measures
Freund-Levi et al. (2008)	Sweden	RCT, double-blind	*N = 204;* 74; male 55%; AD with AChEIs treatment (DSM-IV)	1.7 g DHA and 0.6 g EPA or placebo daily, 26 weeks	NPI, DAD, MADRS	Improvement of NPI in *APOE4* carriers and of MADRS in non-*APOE4* carriers
You et al. (2021)	Malaysia	RCT, double-blind	*N = 48;* range 60 to 75; MCI (Petersen criteria)	*Cosmos caudatus* 500 mg or placebo, daily, 12 weeks	MMSE	Improvement of MMSE
Xu et al. (2012)	China	RCT, double-blind	*N = 78;* 72; male 65%; Mild–Moderate vascular Dementia (DSM-IVR and NINDS-AIREN)	Huperzine A 0.1 mg (BID) or placebo (Vitamin C 100 mg BID), 12 weeks	MMSE, CDR, ADL	Improvement in cognitive measures
Chatzikostopoulos et al. (2024)	Greece	RCT^d^	*N = 80;* 69; male 44%; MCI (DSM-V)	5 drops of Pomegranate Seed Oil or Mediterranean Diet, 52 weeks	MMSE, MoCA, RAVLT, ROCFT, ADAS-cog, TMT B, FUCAS	Improvement of ADAS-cog, RAVLT and TMT B
Fernando et al. (2023)	Sri Lanka	RCT, double-blind	*N = 84;* 73; male 34%; Mild–Moderate AD (NINCDS/ADRDA)	30 mL Virgin Coconut oil or Canola oil (Control) daily, 24 weeks	MMSE, CLOX	No significant differences in cognitive scores. MMSE scores improved among *APOE* ε4 carriers.

ADAS-Cog, Alzheimer’s Disease Assessment Scale-Cognitive Subscale; ADAS-noncog, Alzheimer’s Disease Assessment Scale-Noncognitive Subscale; ADCS-ADL, Alzheimer’s Disease Cooperative Study Activities of Daily Living Inventory 23 - item Scale; CDR-SB, Clinical Dementia Rating Scale – Sums of Boxes; CDR, Clinical Dementia Rating, CDT, Clock Drawing Test; CERAD, Consortium to Establish a Registry for Alzheimer’s Disease Assessment Packet; CFT, Category Fluency Test; CGI (item 2), Clinical Global Impression; CGIC, Clinical Global Impression of Change; CIBIC, Clinician Interview-Based Impression of Change with Caregiver Input; CLOX, Clock Drawing Task; DAD, Disability Assessment for Dementia; DEMQOL, Dementia Quality of Life; FAST, Functional Assessment Staging; FRSSD, Functional Rating Scale of Symptoms of Dementia; FUCAS, Functional Cognitive Assessment Scale; GAI, Geriatric Anxiety Inventory; GDS, Geriatric Depression Scale; GERRI, Geriatric Evaluation by Relative’s Rating Instrument; IADL, Instrumental Activities of Daily Living; MADRS, Montgomery-Asberg Depression Rating Scale; MCI, Mild Cognitive Impairment; MMSE, Mini Mental State Examination; MoCA, Montreal Cognitive Assessment; NIA-AA, National Institute on Aging - Alzheimer’s Association Workgroup; NPI, Neuropsychiatric Inventory; PSQI, Pittsburgh Sleep Quality Index; RAVLT, Rey Auditory Verbal Learning Test; RBANS, Repeatable Battery for the Assessment of Neuropsychological Status; RCFT, Rey Complex Figure Test; SCAG, Sandoz Clinical Assessment - Geriatric; ROCFT, Rey-Osterrieth Complex Figure Test; SCIRS, Severe Cognitive Impairment Rating Scale; SDI, Sleep Disorders Inventory; SIB, Severe Impairment Battery; SKT, Syndrom Kurz test; TMT B, Trail Making Test Part B; VFT, Verbal Fluency Test; VLT, Verbal Learning Test; VR, visual reproduction; WAIS-III, Wechsler Adult Intelligence Scale - III; WATR, Wechsler Test of Adult Reading; WCST-64, Wisconsin Card Sorting Test - 64; WHO-5, World Health Organization 5 Well-Being Index; WHOQOL-BREF, World Health Organization Quality of Life questionnaire; WMS-IV, Memory Scale Fourth Edition; WMS-R, Wechsler Memory Scale – Revised.^a^ Diagnosed, hospitalized, nursing, or cognitive deficit and/or personality change present for at least 6 months, as observable by a physician and/or close contact of the patient, in combination with MMSE, SKT, or CERAD.^b^ Recruited through ADCS.^c^ Dosage not specified.^d^ Blind procedure not reported.

The total sample size across the included studies was 8,532 participants, with a mean age of 72 years. Among these studies, 31 focused on older adults clinically diagnosed with mild to moderate Alzheimer’s disease (AD) based on established diagnostic criteria such as NINCDS-ADRDA, DSM-III, or DSM-IV (e.g., Akhondzadeh et al., 2003a, 2003b; Akhondzadeh et al., 2010a; Akhondzadeh et al., 2010b; Fernando et al., 2023; Freund-Levi et al., 2008; Herrschaft et al., 2012; Hofferberth, 1994; Ihl et al., 2011; Le Bars et al., 1997; Le Bars et al., 2002; Maurer et al., 1997; Mazza et al., 2006; Muangpaisan et al., 2022; Noguchi-Shinohara et al., 2020; Quinn et al., 2010; Rafii et al., 2011; Rasi Marzabadi et al., 2022; Schneider et al., 2005; Thal et al., 1999; Turner et al., 2015; van et al., 2000; Wade et al., 2014; Wang et al., 2018; Xu et al., 2012). Two studies investigated vascular dementia (Erkinjuntti et al., 2002; Xu et al., 2012), and two other examined AD with comorbid neuropsychiatric symptoms (Herrschaft et al., 2012; Ihl et al., 2011). Additional studies addressed participants with multi-infarct dementia (Le Bars et al., 1997; Le Bars et al., 2002) or concurrent use of acetylcholinesterase inhibitors (AChEIs) (Freund-Levi et al., 2008). Twelve studies specifically targeted older adults with MCI (e.g., Boespflug et al., 2018; Calapai et al., 2017; Chatzikostopoulos et al., 2024; Kaddoumi et al., 2022; Lee et al., 2017; Lee et al., 2013; Lee et al., 2020; Noguchi-Shinohara et al., 2023; Park H. et al., 2019; Shin et al., 2009; Tsolaki et al., 2016; Tsolaki et al., 2020; You et al., 2021), and one study focused on amnestic and multi-domain MCI (Tsolaki et al., 2016). Two studies exclusively addressed participants with moderate-to-severe AD (Farlow et al., 2019; Farokhnia et al., 2014).

The interventions in the reviewed studies had an average duration of 27 weeks, ranging from 6 to 96 weeks, and predominantly utilized natural extracts. These extracts included *Cocos nucifera* (*N* = 1; Fernando et al., 2023), *Cosmos caudatus* (*N* = 1; You et al., 2021), *Crocus sativus* L. (*N* = 5; Akhondzadeh et al., 2010a, Akhondzadeh et al., 2010b; Farokhnia et al., 2014; Rasi Marzabadi et al., 2022; Tsolaki et al., 2016), *Ganoderma lucidum* (*N* = 1; Wang et al., 2018), *Garcinia mangostana* L. (*N* = 1; Muangpaisan et al., 2022), *Ginkgo biloba* (*N* = 8; Herrschaft et al., 2012; Hofferberth, 1994; Ihl et al., 2011; Le Bars et al., 1997; Le Bars et al., 2002; Maurer et al., 1997; Mazza et al., 2006; Schneider et al., 2005), *Melissa officinalis* (*N* = 3; Akhondzadeh et al., 2003a; Noguchi-Shinohara et al., 2023; Noguchi-Shinohara et al., 2020), *Olea europaea* L. (*N* = 2; Kaddoumi et al., 2022; Tsolaki et al., 2020), *Panax ginseng* (*N* = 1; Park H. et al., 2019), *Polygala tenuifolia* Willdenow (*N* = 1; Shin et al., 2009), *Punica granatum* (*N* = 1; Chatzikostopoulos et al., 2024), *Salicornia europaea* L. (*N* = 1; Lee et al., 2020), *Salvia officinalis* (*N* = 1; Akhondzadeh et al., 2003b), *Vitis vinifera* (*N* = 2; Calapai et al., 2017; Lee et al., 2017), *Vaccinium ashei*, and *Vaccinium corymbosum* L. (*N* = 1; Boespflug et al., 2018). These supplements were administered orally, primarily in the form of capsule powders, capsule liquids, or liquid solutions, as detailed in Table 1. The concentration of the natural extracts varied significantly across studies. Specifically, *Cocus nucifera* was administered at a dosage of 30 mL per day, *Cosmos caudatus* at 500 mg/day, and *Crocus sativus* L. at 30 mg/day. *Ganoderma lucidum* was provided at 1 g/day, while *Garcinia mangostana* L. dosage ranged from 220 to 560 mg/day depending on body weight. For *Ginkgo biloba*, the standardized *Ginkgo biloba* extract (EGb) 761 was administered in dosages ranging from 80 mg/day to 240 mg/day, typically at 120 or 240 mg/day. *Punica granatum* was given as 5 drops of seed oil daily. The administration of *Melissa officinalis* varied among studies: Noguchi-Shinohara et al. (2023), Noguchi-Shinohara et al. (2020) provided capsule powder containing at least 500 mg of rosmarinic acid per capsule, whereas Akhondzadeh et al. (2003a) offered a liquid solution with a concentration sufficient to provide at least 500 μg of citral per milliliter. The administration protocols for *Olea europaea* L. also differed between studies. Kaddoumi et al. (2022) supplemented participants with 30 mL/day of extra-virgin oil compared to refined olive oil at the same dosage. In contrast, Tsolaki et al. (2020) supplemented participants with high phenolic early extra-virgin olive oil or moderate phenolic oil at 50 mL/day, comparing these interventions to a Mediterranean diet. *Panax ginseng* was supplied in powder from at a dosage of 3 g/day, *Polygala tenuifolia* Willdenow (root extract powder designated as BT-11) at 300 mg/day, and *Salicornia europaea* at 600 mg/day. *Salvia officinalis* was administered as a liquid solution. *Vitis vinifera* was provided as powders at dosages of 250 mg/day (Calapai et al., 2017) and 72 g/day (Lee et al., 2017). Lastly, a combination of *Vaccinium ashei Reade* and *Vaccinium corymbosum* L. was administered in a 1:1 ratio at a daily dose of 25 g (Boespflug et al., 2018).

In addition to these natural extracts, fifteen studies supplemented participants with other natural compounds, including Bryostatin (*N* = 1; Farlow et al., 2019), Docosahexaenoic acid (DHA) (*N* = 3; Freund-Levi et al., 2008; Lee et al., 2013; Quinn et al., 2010), Huperzine A (*N* = 2; Rafii et al., 2011; Xu et al., 2012), Melatonin (*N* = 1; Wade et al., 2014), Physostigmine (*N* = 2; Thal et al., 1999; van et al., 2000), Galantamine (*N* = 5; Erkinjuntti et al., 2002; Raskind et al., 2000; Rockwood et al., 2001; Tariot et al., 2000; Wilcock et al., 2000), and Resveratrol (*N* = 1; Turner et al., 2015). Bryostatin was intravenously infused seven times over a 12-week period, with dosages of either 24 μg twice and 20 μg five times or 48 μg twice and 40 μg five times, and the mean infusion time was 45 ± 5 min. For DHA supplementation, Quinn et al. (2010) used algae-derived DHA without eicosapentaenoic acid (EPA) at 2 g/day, whereas Lee et al. (2013) and Freund-Levi et al. (2008) utilized fish-derived DHA containing EPA, supplementing participants with 1.3 g DHA plus 0.45 g EPA/day and 1.7 g DHA pluls 0.6 g EPA/day, respectively.

Huperzine A was administered at dosages of 0.1 mg, 0.2 mg, or 0.4 mg/day. Galantamine dosages ranged from 8 mg to 32 mg/day, with 24 mg/day being the most frequently used dosage. Resveratrol was administered with an escalating dosage ranging from 500 mg to 2,000 mg/day.

All studies included in the analysis employed a RCT design. Among these, one study implemented a single-blind procedure (Tsolaki et al., 2016), and two studies did not report a blinding procedure (Chatzikostopoulos et al., 2024; Wang et al., 2018). The remaining studies were conducted with double-blinding. Most studies adhered to well-designed case–control methodologies in accordance with predefined inclusion criteria and provided comprehensive descriptions of their objectives, definitions, and methodologies.

While all studies aimed to investigate the effects of natural compounds or extracts on cognitive health, there were variations in the selection of control groups. Specifically, 37 studies utilized isoenergetic placebos as the control intervention, whereas four studies employed commonly prescribed drugs. For instance, Rasi Marzabadi et al. (2022), Akhondzadeh et al. (2010a), and Mazza et al. (2006) used donepezil as the control intervention to compare its effects with those of *Crocus sativus* or *Ginkgo biloba* extract. Farokhnia et al. (2014) used memantine as the control intervention to compare its effects with *Crocus sativus* L. One study on *Olea europaea L.* (Kaddoumi et al., 2022) used refined olive oil as the control to compare with extra-virgin olive oil, while other studies on *Olea europaea L.* and *Punica granatum* employed the Mediterranean diet as the control intervention (Chatzikostopoulos et al., 2024; Tsolaki et al., 2020). Additionally, Fernando et al. (2023) utilized canola oil as the control intervention to compare its effects with those of virgin coconut oil. These methodological considerations, including blinding procedures and the selection of appropriate control groups, were meticulously implemented to ensure the robustness and validity of the findings regarding the impact of natural compounds and extracts on cognitive health.

### 3.3 Study quality

**Figure 2:**
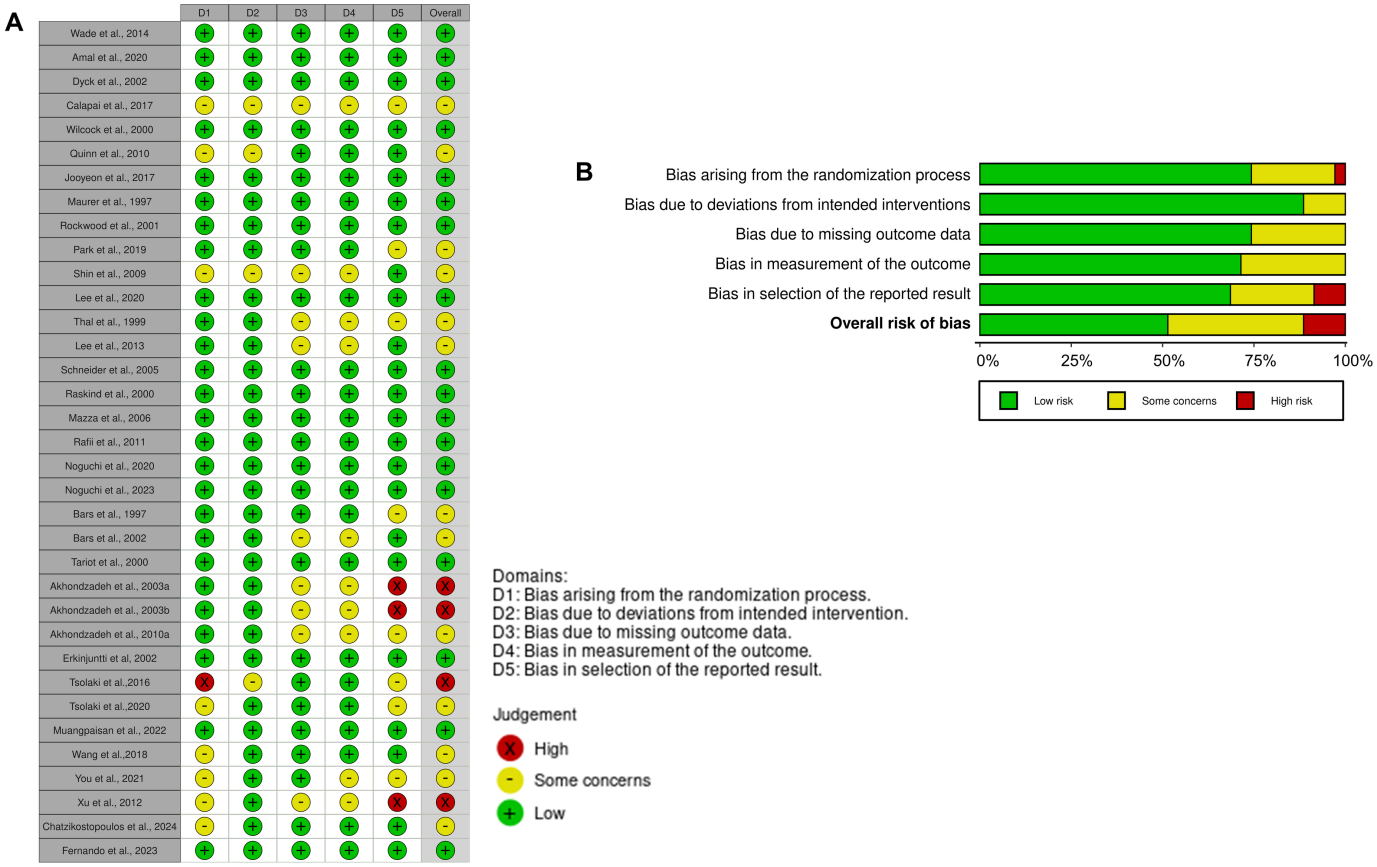
Risk of bias evaluation using Cochrane ROB2. Illustration **(A)** presents the outcomes of bias assessment as per the Cochrane framework, while Illustration **(B)** summarizes these findings. RoB2, *Revised Cochrane risk-of-bias tool for RCTs*.

The quality of the included RCTs was assessed using the Cochrane Risk of Bias tool. Of the 35 studies included in the meta-analysis, 25 exhibited a low risk of bias in randomization, 8 raised some concerns, and 1 was rated as high risk (Figure 2). Deviation from intended interventions showed low risk in 31 studies, while 4 raised concerns. Regarding missing outcome data, 27 studies were rated as low risk and 9 as having some concerns. Outcome measurement risk was low in 25 studies, with 10 raising concerns. Finally, selection bias for reported results was rated low in 24 studies, with 8 raising some concerns and 3 being high risk.

Overall, 18 studies were deemed to have low risk, 13 presented some concerns, and 4 demonstrated high risk of bias (Figure 2). These assessments provided a robust foundation for interpreting the results of the meta-analysis.

### 3.4 Key results from the studies encompassed in the systematic review

Table 1 displays all 45 studies included in the cognitive analysis. Of these, 34 studies reported trends toward cognitive improvement with supplement use, four studies found no significant differences between supplements and commonly prescribed drugs (donepezil, memantine), and seven studies observed no improvement in cognitive measures.

#### 3.4.1 Primary findings from studies on natural extracts

Fernando et al. (2023) conducted a 24-week study involving patients with MCI who received 30 mL of virgin coconut oil (VCO) daily. While overall supplementation with VCO did not result in significant cognitive improvements, patients carrying the APOE ε4 allele exhibited enhanced MMSE scores compared to controls. The intervention was deemed safe, as lipid profiles and glycated hemoglobin levels remained stable. Similarly, administration of *Cosmos caudatus* for 12 weeks led to significant enhancements in cognitive and mood-related outcomes, including MMSE scores, tension, mood disturbance, and malondialdehyde levels. However, You et al. (2021) noted that the short duration and poor bioavailability of flavonoids might limit the biochemical effects of *Cosmos caudatus*.

Several studies on *Crocus sativus* L. (also known as saffron) reported cognitive benefits. Notably, one-year supplementation resulted in magnetic resonance imaging (MRI)-detected structural changes in the left inferior temporal gyrus, potentially linked to improved cognitive function. Electroencephalogram (EEG) assessments revealed shorter P300 latencies, indicating enhanced cognitive processing speed. In a trial by Akhondzadeh et al. (2010a), saffron supplementation for up to 16 weeks improved attention, memory, and visual-motor coordination in patients with mild-to-moderate AD, as evidenced by higher scores on the ADAS-Cog and clinical dementia rating sum of boxes (CDR-SB). Conversely, Farokhnia et al. (2014) compared *Crocus sativus* with memantine in moderate-to-severe AD patients over one year. Both treatments attenuated cognitive decline, with saffron effectively reducing behavioral and psychological symptoms of dementia. The intervention was well-tolerated, with only mild, self-limiting gastrointestinal symptoms, dizziness, and headaches reported. In contrast, supplementation with *Ganoderma lucidum* did not yield significant cognitive or quality of life improvements over a six-week period in a study involving 42 AD patients, likely due to the short intervention duration and small sample size. On the other hand, *Garcinia mangostana L.* supplementation for up to 24 weeks demonstrated significant improvements in ADAS-cog scores and reductions in the oxidative stress biomarker 4-hydroxynonenal in a low-dose group, with the intervention being well-tolerated (Muangpaisan et al., 2022).

*Ginkgo biloba* has been extensively studied, yielding mixed results. Schneider et al. (2005) found no significant difference in ADAS-cog scores between the treatment and placebo groups after 52 weeks in patients with mild-to-moderate AD. However, at the 26-week mark, clinician’s interview-based impression of change plus (CIBIC+) scores improved significantly in the treatment group. Le Bars et al. (1997) reported improvements in ADAS-Cog score and the Geriatric evaluation by relative’s rating instrument (GERRI) in *Ginkgo biloba*-treated subjects compared to placebo. Additionally, Herrschaft et al. (2012) demonstrated that *Ginkgo biloba* improved cognition, psychopathology, functional measures, and quality of life in patients with mild-to-moderate dementia, including AD and vascular dementia, over a 24-week period. The supplementation of *Ginkgo biloba* in older adults warrants caution due to its potential to increase bleeding risk when combined with anticoagulants or antiplatelet agents (Ke et al., 2021) and to disrupt blood glucose regulation in diabetic patients with AD (Kudolo, 2001). Consequently, caregivers contemplating the use of natural products or extracts are strongly advised to seek guidance from healthcare professionals. Furthermore, healthcare providers should thoroughly assess the potential adverse effects of such supplements before recommending them, particularly in the clinical management of individuals with MCI or AD who often present with complex comorbidities.

*Melissa officinalis* (also known as lemon balm) supplementation showed cognitive benefits in AD patients. A 16-week study administering 500 μg per day improved ADAS-cog and CDR-SB scores and reduced agitation in patients with mild-to-moderate AD. Longer trials (96 weeks) with 500 mg of rosmarinic acid per day suggested potential preventative effects on cognitive decline in non-hypertension subjects. Tsolaki et al. (2020) investigated high-phenolic and moderate-phenolic extra-virgin olive oil (EVOO) and reported significant improvements in most cognitive domains compared to a Mediterranean diet. Complementary studies have linked EVOO consumption to reduced blood–brain barrier permeability in brain regions associated with memory and cognitive performance (Kaddoumi et al., 2022).

In Korean subjects with MCI, six months of *Panax ginseng* supplementation improved visual memory (Park H. et al., 2019). *Polygala tenuifolia* extract enhanced word recognition and recall and improved the overall scores in a mental cognitive test battery in aging adults, showing effects comparable to placebo (Shin et al., 2009; Wang et al., 2019). *Punica granatum* seed oil supplementation over 52 weeks benefited cognitive functions, as indicated by ADAS-Cog and memory tests, with pre- to post-treatment improvements in processing and executive functions in the MCI group (Chatzikostopoulos et al., 2024).

Additional interventions included *Vitis vinifera*, which improved cognitive and mood scores over 12 weeks in elderly individuals (Calapai et al., 2017), and *Vaccinium* (blueberry) supplementation in MCI, which was associated with increased activation in brain regions involved in memory (Boespflug et al., 2018). A phase II study on Bryostatin reported no overall significant effect on severe impairment battery (SIB) scores; however, there were positive cognitive trends in completers at 20 μg (7 doses/12 weeks), although higher doses led to dropouts due to adverse events (Farlow et al., 2019).

DHA supplementation yielded varied outcomes. Quinn et al. (2010) did not observe a reduction in the rate of cognitive decline in AD patients, whereas Lee et al. (2013) found that fish-derived DHA containing EPA enhanced short-term and working memory in elderly subjects with MCI. Freund-Levi et al. (2008) reported that DHA supplementation in AD patients decreased agitation and depression independent of cognitive improvement. Collectively, these studies suggest that various natural supplements may offer symptomatic and cognitive benefit in both MCI and AD.

#### 3.4.2 Major findings of studies on natural compounds

Recent trials have explored diverse pharmacological approaches to address cognitive decline in both AD and vascular dementia. Huperzine A has shown significant potential as a cognitive enhancer. A 16-week phase II trial in patients with mild-to-moderate AD demonstrated its safety and tolerability, with statistically significant cognitive improvements compared to placebo as measured by the MMSE and ADAS-cog (Rafii et al., 2011). Similarly, a 12-week study on vascular dementia patients revealed significant cognitive enhancements on the MMSE, Clinical Dementia Rating (CDR), and Activities of Daily Living (ADL) scales, with greater gains observed in the treatment group (Xu et al., 2012). Gastrointestinal symptoms, including nausea, vomiting, and diarrhea, were the most commonly reported adverse events, though they were generally mild and transient.

Prolonged-release melatonin (PRM) has also emerged as a promising add-on therapy for AD. Wade et al. (2014) found that PRM significantly improved cognitive performance, as evidenced by MMSE and Instrumental Activities of Daily Living (IADL) scores, and enhanced sleep quality based on the Pittsburgh Sleep Quality Index. These effects were particularly notable in patients with comorbid insomnia, who exhibited clinically meaningful improvements in cognition and sleep efficiency compared to placebo. PRM was well-tolerated, with an adverse event profile comparable to the placebo group (Wade et al., 2014).

Physostigmine has been evaluated for cognitive enhancement in mild-to-moderate AD with mixed outcomes. van et al. (2000) reported significant improvements in ADAS-cog and Clinician’s Interview-Based Impression of Change Plus (CIBIC+) scores, but no benefits were observed in secondary outcomes such as the Clinical Global Impression of Change (CGIC). Additionally, gastrointestinal side effects, including nausea and vomiting, were prevalent, affecting 47% of participants and limiting its usability. Similarly, Thal et al. (1999) observed significant cognitive and behavioral improvements with controlled-release physostigmine but noted high dropout rates due to adverse gastrointestinal effects, such as nausea, diarrhea, and dyspepsia. Despite these limitations, both studies indicated an acceptable safety profile, with no cardiac rhythm disturbances or liver function abnormalities reported.

Galantamine, a cholinesterase inhibitor, has consistently demonstrated robust efficacy in improving cognitive and functional outcomes in AD across multiple studies. Rockwood et al. (2001) reported superior cognitive performance ADAS-cog and global response rates (CIBIC+) compared to placebo over three months, with fewer patients experiencing cognitive decline. Galantamine also enhanced both basic and instrumental ADL while maintaining a favorable tolerability profile, with gastrointestinal symptoms being the most frequent but generally mild. Long-term efficacy was confirmed in studies by Raskind et al. (2000) and Wilcock et al. (2000), which demonstrated sustained improvements in cognition, daily functioning, and clinician-rated impressions of change over six months. Notably, slow dose escalation strategies improved tolerability and reduced adverse events. These findings underscore the therapeutic potential of galantamine in managing AD symptoms.

Resveratrol, a naturally occurring polyphenol, has produced less definitive clinical outcomes. In a year-long trial involving AD patients, Turner et al. (2015) observed no statistically significant effects on AD biomarkers or cognitive function. However, trends toward reductions in cerebrospinal fluid (CSF) Aβ40 levels and increases in the Aβ40/Aβ42 ratio suggested potential effects on amyloid deposition. Resveratrol was generally well-tolerated, with no significant differences in adverse events between treatment and placebo groups. The study’s limited sample size and duration, however, constrained the generalizability of its findings.

In summary, Huperzine A, PRM, physostigmine, and galantamine have demonstrated varying degrees of efficacy as cognitive enhancers in AD and vascular dementia, with galantamine emerging as a particularly promising option due to its sustained benefits and tolerability. While resveratrol holds theoretical potential, its clinical utility remains inconclusive, necessitating further investigation into its long-term effects and mechanisms of action.

### 3.5 Meta-analyses

The cognitive assessments employed in the included studies utilized a variety of tools, such as the ADAS-cog, MMSE, CDR-SB, Disability Assessment for Dementia, Functional Rating Scale of Symptoms of Dementia, and Geriatric Depression Scale (Table 1). However, not all of these measures were incorporated into the subsequent meta-analyses.

**Figure 3:**
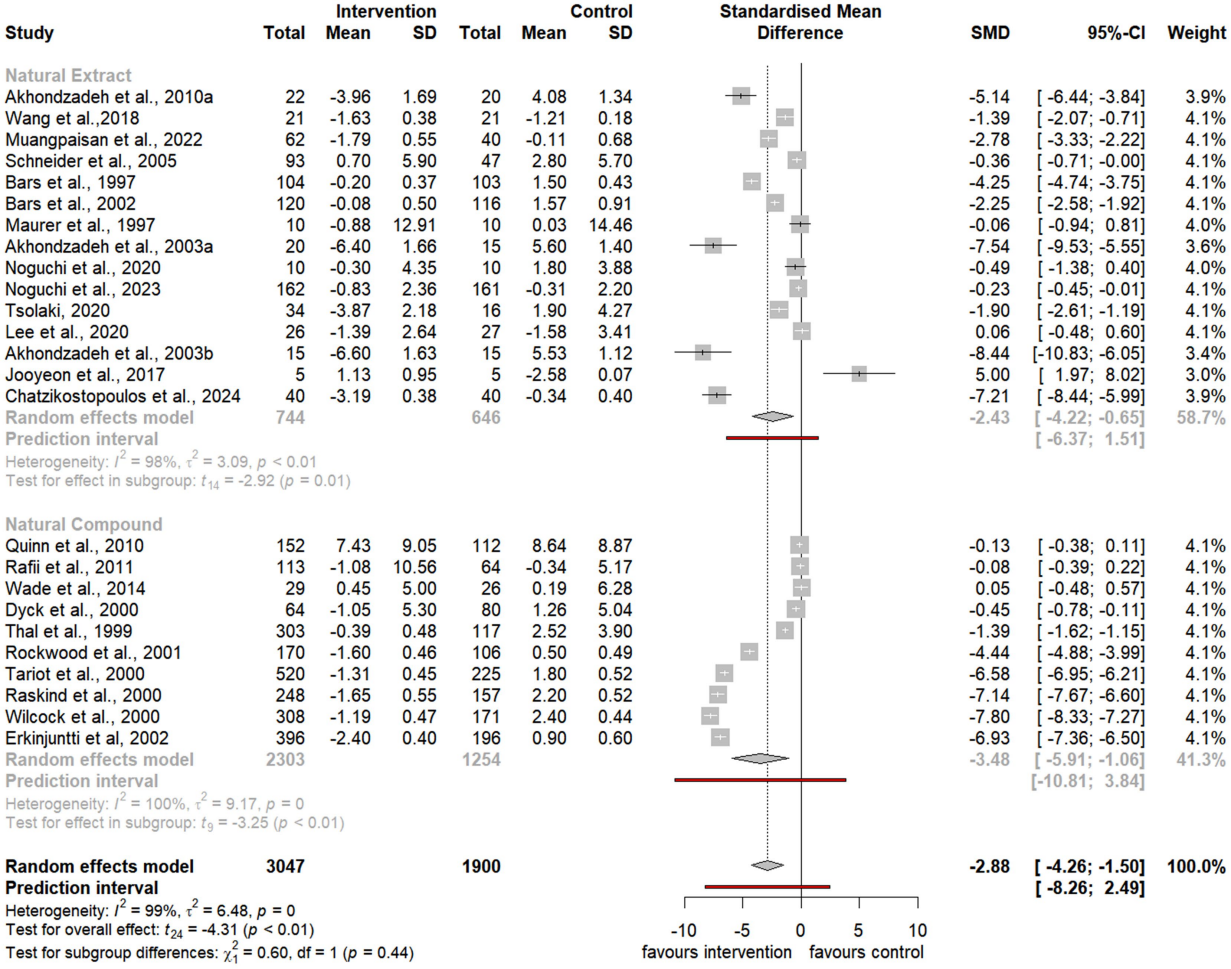
Forest plot for effect of natural compound/extract intervention studies assessing ADAS-cog classified by type of supplementation. ADAS-cog, *Alzheimer Disease Cooperative Study-Activities of Daily Living Scale*.

**Figure 4:**
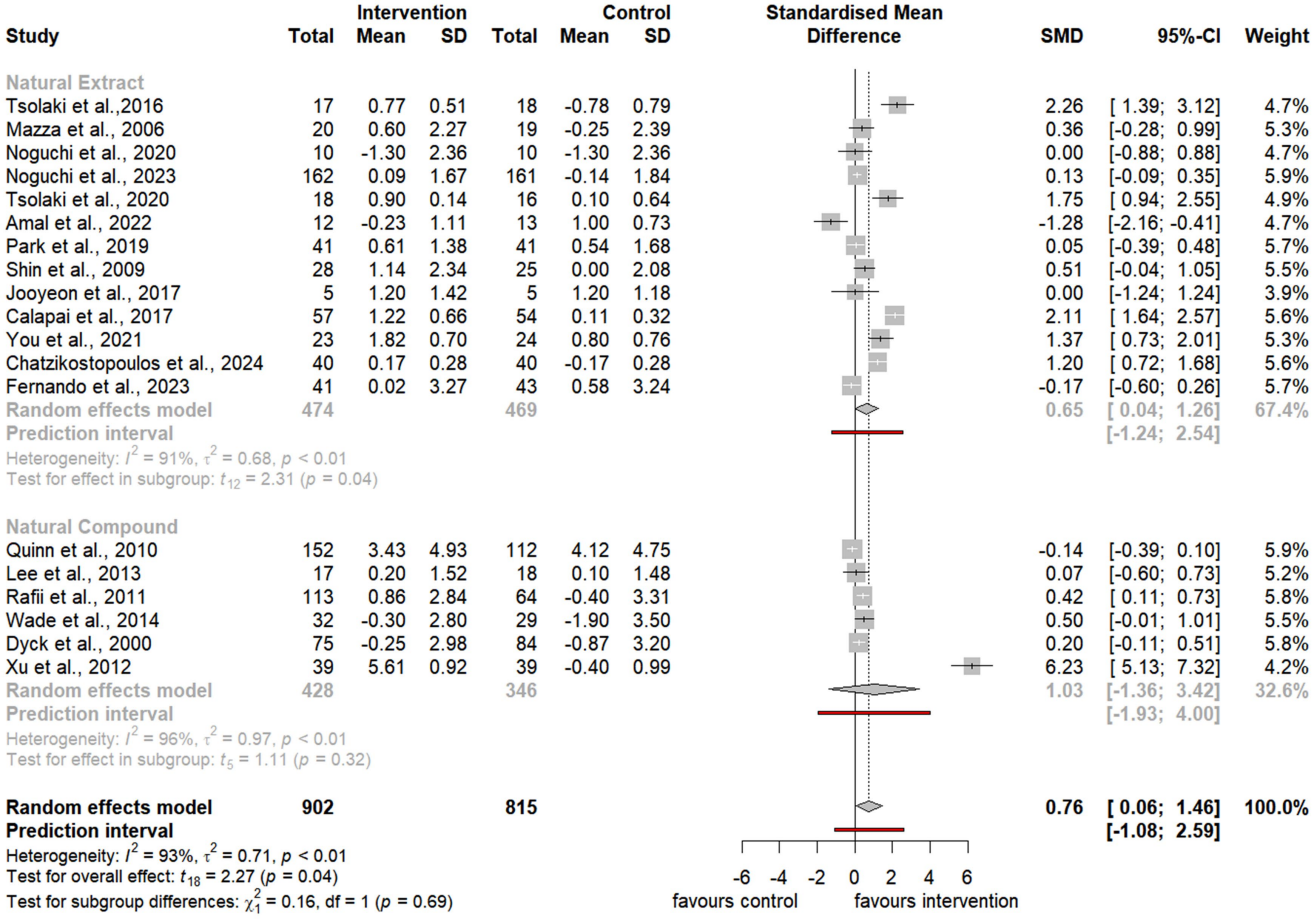
Forest plot for effect of natural compound/extract intervention studies assessing MMSE classified by type of supplementation. MMSE, *Mini Mental State Examination*.

**Figure 5:**
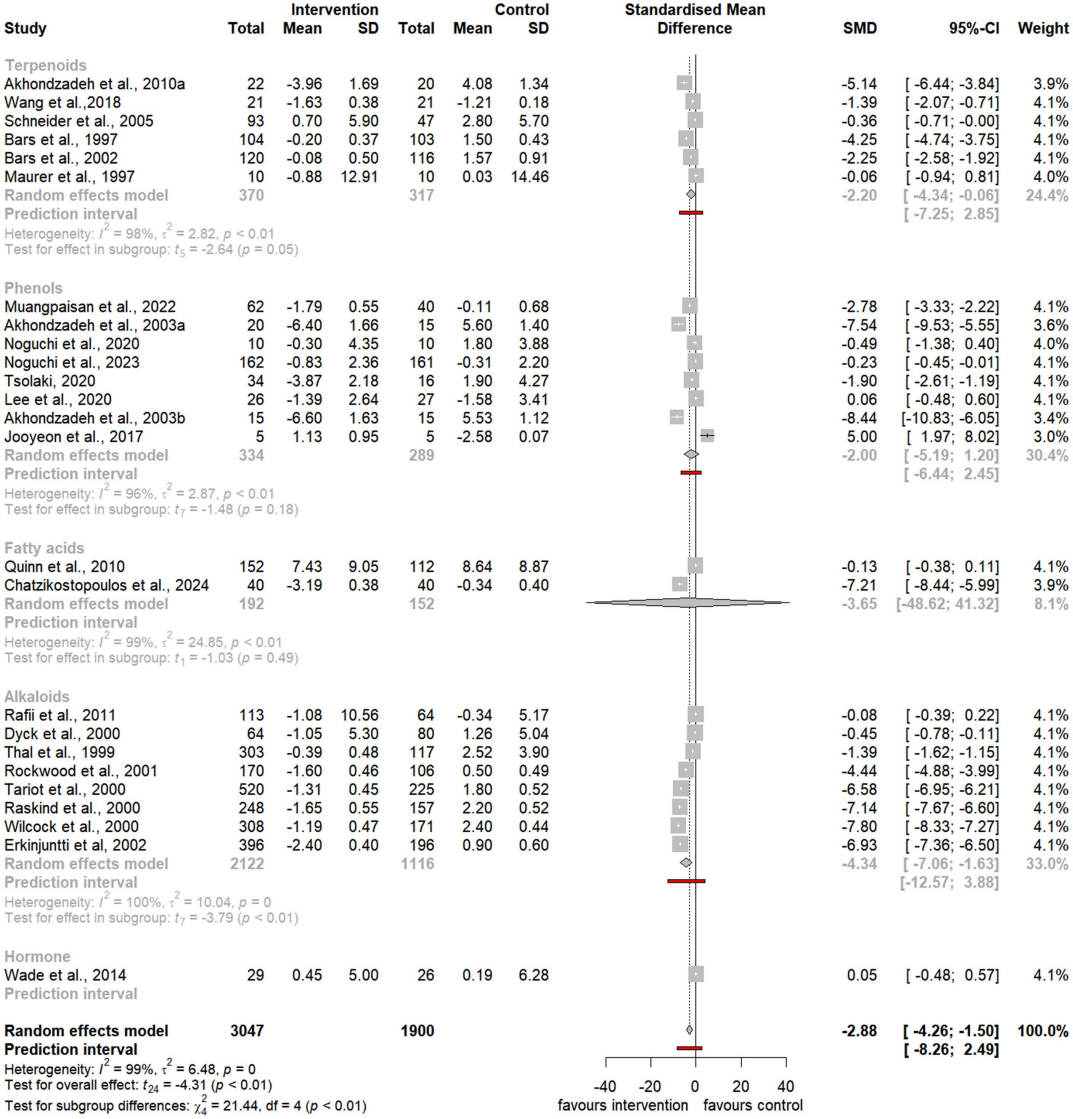
Forest plot for effect of natural compound/extract intervention studies assessing ADAS-cog classified by class of major compound(s). ADAS-cog, *Alzheimer Disease Cooperative Study-Activities of Daily Living Scale*.

**Figure 6:**
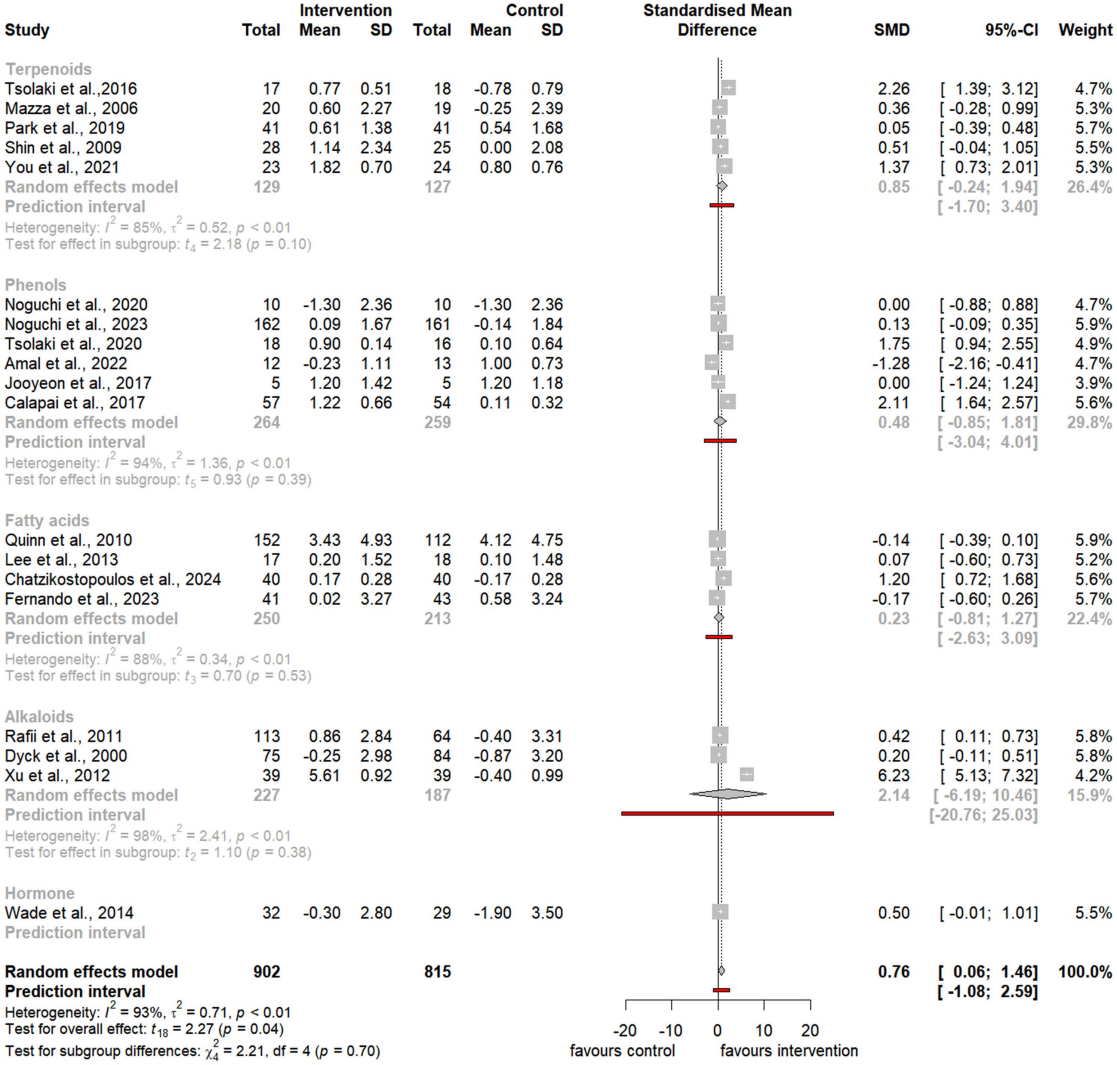
Forest plot for effect of natural compound/extract intervention studies assessing MMSE classified by class of major compound(s). MMSE, *Mini Mental State Examination*.

To assess the overall effect size, the meta-analysis also conducted subgroup analyses to examine the impact of distinct categories, including natural extracts, natural compounds, and specific compound classes such as terpenoids, phenols, and alkaloids (Figures 3–6). However, due to limited data availability, more granular subgroup analyses focusing on specific plant structural extracts (Supplementary Figures S1, S2) and hormonal compounds (Figures 5, 6) could not sufficiently explore variations arising from study designs and participant characteristics. Consequently, these findings must be interpreted with caution and in consideration of the underlying limitations.

As summarized in Table 1, 25 studies provided sufficient data for meta-analysis using the ADAS-cog, a widely recognized neuropsychological tool for evaluating cognitive severity in dementia (Akhondzadeh et al., 2003a, 2003b; Akhondzadeh et al., 2010a; Akhondzadeh et al., 2010b; Chatzikostopoulos et al., 2024; Erkinjuntti et al., 2002; Le Bars et al., 1997; Le Bars et al., 2002; Lee et al., 2017; Lee et al., 2020; Maurer et al., 1997; Muangpaisan et al., 2022; Noguchi-Shinohara et al., 2023; Noguchi-Shinohara et al., 2020; Quinn et al., 2010; Rafii et al., 2011; Raskind et al., 2000; Rockwood et al., 2001; Schneider et al., 2005; Tariot et al., 2000; Thal et al., 1999; Tsolaki et al., 2020; van et al., 2000; Wade et al., 2014; Wang et al., 2018; Wilcock et al., 2000). Additionally, 19 studies provided sufficient data for meta-analysis using the MMSE, a commonly used tool to evaluate cognitive impairment in clinical and research contexts (Calapai et al., 2017; Chatzikostopoulos et al., 2024; Fernando et al., 2023; Kaddoumi et al., 2022; Lee et al., 2017; Lee et al., 2013; Mazza et al., 2006; Noguchi-Shinohara et al., 2023; Noguchi-Shinohara et al., 2020; Park K. C. et al., 2019; Quinn et al., 2010; Rafii et al., 2011; Shin et al., 2009; Tsolaki et al., 2016; Tsolaki et al., 2020; van et al., 2000; Wade et al., 2014; Xu et al., 2012; You et al., 2021).

Notably, the meta-analysis identified a significant improvement in ADAS-cog scores among participants receiving natural extracts or compounds compared to controls (SMD = −2.88, 95% CI −4.26 to −1.50, t_24_ = −4.31, *p* < 0.01) (Figure 3). Similarly, MMSE scores showed significant improvement following interventions compared to controls (SMD = 0.76, 95% CI 0.06 to 1.46, t_18_ = 2.27, *p* = 0.04) (Figure 4).

Despite the observed heterogeneity, these findings suggest that natural extracts and compounds exert a significant and substantial effect on global cognitive function. However, the variability in effect sizes across individual studies indicates that these impacts may differ depending on the cognitive domain or compound characteristics.

To address the heterogeneity, additional subgroup and moderator analyses were conducted to investigate the influence of factors such as the type of natural extract, compound class (e.g., terpenoids, phenols, and alkaloids), and outcome measures on the observed cognitive improvements following intervention.

#### 3.5.1 Subgroup analyses: effects of natural extracts and natural compounds

Subgroup analyses of natural extracts indicated a significant improvement in ADAS-cog scores following supplementation (SMD = −2.43, 95% CI −4.22 to −0.65, t_14_ = −2.92, *p* = 0.01) (Figure 3), although substantial heterogeneity was observed (*I^2^* = 98%, *p* < 0.01). Additionally, there was a notable trend toward improvement in MMSE scores after supplementation (SMD = 0.65, 95% CI 0.04 to 1.26, t_12_ = 2.31, *p* = 0.04), also accompanied by significant heterogeneity (*I^2^* = 91%, *p* < 0.01) (Figure 4).

Similarly, subgroup analyses of natural compounds demonstrated a significant improvement in ADAS-cog scores following supplementation, with a larger effect size (SMD = −3.48, 95% CI −5.91 to −1.06, t_9_ = −3.25, *p* < 0.01) (Figure 3), albeit with considerable heterogeneity (*I^2^* = 100%, *p* = 0). Furthermore, while there was a trend toward improvement in MMSE scores after supplementation, this was not statistically significant (SMD = 1.03, 95% CI −1.36 to 3.42, t_5_ = 1.11, *p* = 0.32) and was accompanied by notable heterogeneity (*I^2^* = 97%, *p* < 0.01) (Figure 4).

#### 3.5.2 Subgroup analyses: effects of terpenoids, phenols, and alkaloids

Subgroup analyses were conducted to evaluate the effects of terpenoids, phenols, and alkaloids. Natural extracts were classified based on the primary bioactive compound classes that have demonstrated efficacy in preclinical or clinical studies related to AD. However, it is essential to note that other bioactive compounds, beyond these primary classes, may also contribute to the observed health benefits. Therefore, the results of these subgroup analyses should be interpreted with caution and careful consideration.

Subgroup analyses focusing on terpenoids revealed a borderline significant improvement in ADAS-cog scores following supplementation (SMD = −2.20, 95% CI −4.34 to −0.06, t_5_ = −2.65, *p* = 0.05) (Figure 5), although substantial heterogeneity was observed (*I^2^* = 98%, *p* < 0.01). Additionally, there was a trend toward improvement in MMSE scores after supplementation (SMD = 0.85, 95% CI −0.24 to 1.94, t_4_ = 2.18, *p* = 0.10), also accompanied by significant heterogeneity (*I^2^* = 85%, *p* < 0.01) (Figure 6).

Similarly, subgroup analyses for alkaloids demonstrated a significant improvement in ADAS-cog scores following supplementation, with a larger effect size (SMD = −4.34, 95% CI −7.06 to −1.63, t_7_ = −3.79, *p* < 0.01) (Figure 5). However, substantial heterogeneity was present (*I^2^* = 100%, *p* = 0). Furthermore, there was a trend toward improvement in MMSE scores, although it was not statistically significant (SMD = 2.14, 95% CI −6.19 to 10.46, t_2_ = 1.10, *p* = 0.38), and notable heterogeneity was present (*I^2^* = 98%, *p* < 0.01) (Figure 6).

Last, subgroup analyses for phenols indicated a trend toward improvement in ADAS-cog scores following supplementation (SMD = −2.00, 95% CI −5.19 to 1.20, t_7_ = −1.48, *p* = 0.18) (Figure 5). However, considerable heterogeneity was observed (*I^2^* = 96%, *p* < 0.01). Similarly, there was a trend toward improvement in MMSE scores, which was not statistically significant (SMD = 0.48, 95% CI −0.85 to 1.81, t_5_ = 0.93, *p* = 0.39), with substantial heterogeneity (*I^2^* = 94%, *p* < 0.01) (Figure 6).

To evaluate potential publication bias, funnel plots (Supplementary Figures S3, S4) were generated to visually examine the distribution of effect sizes. The plots revealed a wide range of effect sizes. Further analysis using Egger’s regression test indicated no significant publication bias (Supplementary Figures S5, S6). Specifically, funnel plots for studies assessing ADAS-cog scores displayed a symmetrical distribution (Supplementary Figure S3), and Egger’s test confirmed the absence of publication bias (Supplementary Figure S5). For MMSE scores, the funnel plots showed an asymmetric distribution (Supplementary Figure S4), but Egger’s regression test was non-significant (Supplementary Figure S6), suggesting no strong evidence of publication bias.

To address potential bias, the Trim-and-Fill method was applied. This adjustment did not significantly alter the findings, further supporting the robustness of the results despite the observed heterogeneity in the studies assessing ADAS-cog and MMSE outcomes.

## 4 Discussion

This systematic review comprehensively analyzed 43 studies evaluating the effects of various natural compounds and extracts, administered in forms such as powders and liquid capsules, as interventions for individuals with MCI or AD. Of these studies, 33 reported significant improvements in cognitive function, while 4 found no notable differences between the natural supplements and conventional pharmacological treatments. The accompanying meta-analysis revealed statistically significant improvements in ADAS-Cog scores in intervention groups compared to controls following supplementation with natural compounds or extracts. Furthermore, a borderline improvement was observed in MMSE scores.

These findings suggest that the analyzed natural compounds, particularly in powder form, may exhibit cognitive-protective properties, although they are unlikely to halt disease progression. The meta-analysis highlights the potential benefits of prolonged supplementation (≥6 weeks) with specific natural compounds or extracts for cognitive enhancement in individuals with MCI or AD. Notably, terpenoids and alkaloids demonstrated superior efficacy in improving global cognitive function compared to phenolic compounds.

### 4.1 Terpenoids

Terpenoids derived from a variety of natural sources, including *Ginkgo biloba*, *Crocus sativus* L. (saffron), ginseng, *Polygala tenuifolia* Willdenow (Polygala), *Ganoderma lucidum* (Reishi mushroom), and *Cosmos caudatus*, exhibit diverse neuroprotective properties and hold therapeutic potential for AD. In *Ginkgo biloba*, terpenoid components such as ginkgolides and bilobalide are recognized for their antioxidant and neuroprotective effects. Although some studies report modest cognitive benefits in AD patients, others find no significant effects. These inconsistencies may stem from variability in formulations, dosages, study designs, and patient-specific factors, including the stage of the disease (DeKosky et al., 2008; Herrschaft et al., 2012; Hofferberth, 1994; Ihl et al., 2011; Kanowski and Hoerr, 2003; Le Bars et al., 1997; Le Bars et al., 2002; Maurer et al., 1997; Mazza et al., 2006; Schneider et al., 2005; Shi et al., 2010; Snitz et al., 2009).

Saffron contains active compounds such as crocin, crocetin, and safranal, which demonstrate antioxidant, anti-inflammatory, and neuroprotective effects. Specifically, crocetin has been shown to modulate A*β* pathology, while safranal enhances cognitive function in preclinical AD models (Ahmad et al., 2023; Akhondzadeh et al., 2010a; Farokhnia et al., 2014; Finley and Gao, 2017; Pandey et al., 2020; Rasi Marzabadi et al., 2022; Tsolaki et al., 2016). Similarly, ginsenosides―the triterpene saponins found in ginseng―exhibit efficacy in improving cognitive function and mitigating AD-related pathologies through their antioxidant and anti-inflammatory properties (Heo et al., 2012; Lee et al., 2008; Park H. et al., 2019; Wang et al., 2019). Tenuifolin, a triterpenoid saponin from *Polygala tenuifolia*, along with related compounds such as polygalasaponin F, has been associated with memory enhancement and neuroprotection by modulating neurotransmitter levels and reducing oxidative stress (Deng et al., 2020; Jia et al., 2004; Moratalla-López et al., 2019; Park H. et al., 2019). In *Ganoderma lucidum*, triterpenoids like ganoderic acids provide neuroprotective effects by attenuating neuroinflammation and oxidative stress, potentially leading to improved cognitive function in AD (Qi et al., 2021; Zheng et al., 2023). Overall, the diverse terpenoid compounds from these natural sources offer promising avenues for the development of therapeutic strategies targeting the multifaceted pathologies of AD.

### 4.2 Phenols

The bioactive constituents of *Cosmos caudatus* include flavonoids (e.g., quercetin and kaempferol), phenolic acids (e.g., caffeic acid), and carotenoids (e.g., β-carotene). These compounds exhibit antioxidant and neuroprotective properties, suggesting their potential in mitigating AD-related neurodegeneration, despite the limited specific research available (Wang et al., 2023). Phenolic compounds found in *Melissa officinalis* (lemon balm), *Olea europaea* (olive), *Garcinia mangostana* (mangosteen), *Salicornia europaea* (samphire), *Salvia officinalis* (sage), and *Vitis vinifera* (grape) further highlight their therapeutic potential. Rosmarinic acid in lemon balm has demonstrated both anti-inflammatory and neuroprotective effects (Petrisor et al., 2022). Compounds derived from olives, such as oleuropein, hydroxytyrosol, and oleocanthal, have shown efficacy in reducing oxidative stress and Aβ pathology while providing cognitive benefits in AD models (Abdallah et al., 2022; Abuznait et al., 2013; Nardiello et al., 2018). Xanthones from mangosteen, including *α*-mangostin and *γ*-mangostin, possess strong antioxidant and anti-inflammatory properties, and catechin flavonoids provide additional neuroprotection (Do and Cho, 2020; Pratiwi et al., 2022; Yang et al., 2021). *Salicornia europaea* contains phenolics and carotenoids, such as lutein, which may help reduce oxidative stress (Fitzner et al., 2021). Sage’s phenolic compounds, including carnosic and ursolic acid, have been shown to improve cognitive function and memory while providing neuronal protection (Ghorbani and Esmaeilizadeh, 2017; Mirza et al., 2021; Yi-Bin et al., 2022). Compounds in grapes, particularly resveratrol, have been found to inhibit Aβ aggregation and neuroinflammation, along with providing additional antioxidant benefits through proanthocyanidins and flavonoids such as quercetin (Tabeshpour et al., 2018).

### 4.3 Alkaloids

Several alkaloids, including bryostatin, huperzine A, physostigmine, and galantamine, exhibit significant therapeutic potential. Bryostatin, a macrolide derived from *Bugula neritina*, modulates protein kinase C and has shown promise in treating neurodegenerative and oncological conditions (Farlow et al., 2019; Nelson et al., 2017; Zonder et al., 2001). Huperzine A, extracted from *Huperzia serrata*, enhances cognitive function by inhibiting acetylcholinesterase, a key enzyme involved in AD pathology (Friedli and Inestrosa, 2021; Liu et al., 2020; Rafii et al., 2011; Xu et al., 2012). Galantamine, an FDA-approved acetylcholinesterase inhibitor, alleviates AD symptoms by enhancing cholinergic signaling, thereby providing cognitive and functional benefits (Santos et al., 2020).

### 4.4 Other classes

Other bioactive compounds, such as omega-3 fatty acids (e.g., DHA) and melatonin, contribute significantly to neuroprotection in AD. DHA has been shown to reduce neuroinflammation, oxidative stress, and Aβ aggregation, while also supporting neuronal survival and synaptic plasticity (Freund-Levi et al., 2008; Khalid et al., 2022; Lee et al., 2013; Quinn et al., 2010; Thomas et al., 2015; Xiao et al., 2022; Yurko-Mauro et al., 2010). Melatonin, known for its role in regulating circadian rhythms and sleep, provides antioxidant and anti-inflammatory effects that mitigate AD pathology, including Aβ accumulation and tau hyperphosphorylation (Cardinali et al., 2014; Furio et al., 2007; Hardeland, 2018; Li et al., 2020; Lin et al., 2013; Wade et al., 2014; Xu et al., 2020).

These natural compounds present a multifaceted approach to combating AD by targeting oxidative stress, inflammation, and amyloid and tau pathologies. However, further research is required to optimize their clinical utility and establish standardized protocols for therapeutic application.

### 4.5 Strengths and limitations

The meta-analysis presented in this study emphasizes the need for further research to validate the potential cognitive benefits of natural compounds and extracts, despite the promising results identified in individual interventions. While previous systematic reviews have addressed natural compounds in preclinical and clinical trials (Ahmad et al., 2023; Andrade et al., 2019; Li et al., 2023), this study distinguishes itself by focusing exclusively on RCTs that evaluated global cognitive domains using widely accepted measures such as the ADAS-cog and MMSE. By employing meta-analyses and providing statistical evidence, this study contributes to a more comprehensive understanding of the effects of natural compounds and extracts. Furthermore, the study’s selection criteria targeted RCTs that utilized a single species of natural extract or specific compound, excluding those involving multiple extracts, compounds, or formulations. This approach aligns closely with real-world practices typically employed by caregivers, making the findings highly relevant for daily clinical management of individuals with MCI or AD. It is essential for individuals considering any dietary supplement to consult healthcare professionals or AD specialists, who can provide personalized guidance based on individual circumstances and health status. Although short-term cognitive improvement was observed across all RCTs, the validation of long-term efficacy in individuals with cognitive impairment necessitates large-scale longitudinal trials. To the best of our knowledge, this study is the first systematic review and meta-analysis that compares the effects of various forms of natural compounds and extracts on patients with MCI or AD using recognized assessment measures such as the MMSE and ADAS-cog. Despite the positive findings, several limitations must be acknowledged. This include the diversity in intervention types, variations, in duration and participant characteristics, and the moderate to high risk of bias in the quality of studies using the same cognitive tasks. Additionally, the vast scope of this topic inevitably meant that not all types or species of natural compounds and extracts could be included. Some natural compounds are still in the preclinical or early clinical phases, and certain studies employing non-RCT designs, such as pilot or cross-over studies, were excluded from this review. This exclusion does not imply a lack of potential benefit to cognitive health. Despite a rigorous search methodology, including additional hand-searching, it is possible that some relevant articles were inadvertently missed. Furthermore, each natural compound and extract originates from distinct sources and possesses unique mechanisms of action and biological effects, necessitating a cautious interpretation of the findings. Future research should address these limitations and aim to provide a more comprehensive understanding of the therapeutic potential of natural compounds and extracts in improving cognitive function.

## 5 Conclusion

This systematic review provides preliminary evidence suggesting the potential cognitive benefits of natural compounds and extracts, particularly as assessed by the ADAS-cog. Additionally, there is significant suggestive evidence indicating improvements in MMSE scores. Notably, this study represents the first systematic review and meta-analysis to comprehensively compare and categorize the effects of various forms of prolonged consumption of natural compounds, extracts, and isolated food supplements in individuals diagnosed with MCI or AD. The study does not offer robust evidence to endorse any individual natural compound or extract reviewed as a substitute for conventional medications in the prevention or treatment of mild cognitive impairment or Alzheimer’s disease.

## Data Availability

The original contributions presented in the study are included in the article/Supplementary material, further inquiries can be directed to the corresponding author.
