# Supplementary material for: Improving cognitive impairment through chronic consumption of natural compounds/extracts: a systematic review and meta-analysis of randomized controlled trials
Source: Front Aging Neurosci. 2025 Jan 30;16:1531278. doi: 10.3389/fnagi.2024.1531278 (PMC11821934; doi:10.3389/fnagi.2024.1531278)
Supplement: Supplementary file 1 [file Table_1.docx]

**Supplemental table 1.** PICOS framework

| **Population** | Adult subjects (≥ 18 years of age) |
| --- | --- |
| **Intervention** | Studies of interventions with a duration of at least six weeks and that involve the provision or promotion of natural compounds/extracts in different forms such as powders, liquids, or capsules for consumption |
| **Comparator** | Control groups that do not receive any natural compounds/extracts and are likely to be given an isoenergetic placebo |
| **Outcome** | Assessments of cognitive function using cognitive tasks and scales that are global in nature |
| **Study design** | Randomised controlled trials |

**Supplemental table 2.** PRISMA CHECKLIST

| **Section and topic** | **Item #** | **Checklist item** | **Location where item is reported** |
| --- | --- | --- | --- |
| **TITLE** | | | |
| Title | 1 | Identify the report as a systematic review. | Page 01 |
| **ABSTRACT** | | | |
| Abstract | 2 | See the PRISMA 2020 for Abstract checklist. | Page 01 |
| **INTRODUCTION** | | | |
| Rationale | 3 | Describe the rationale for the review in the context of existing knowledge. | Page 02 |
| Objectives | 4 | Provide an explicit statement of the objective(s) or question(s) the review addresses. | Page 02 |
| **METHOD** | | | |
| Eligibility criteria | 5 | Specify the inclusion and exclusion criteria for the review and how studies were grouped for the syntheses. | Page 02 |
| Information sources | 6 | Specify all databases, registers, websites, organisations, reference lists, and other sources searched or consulted to identify studies. Specify the date when each source was last searched or consulted. | Page 02 |
| Search strategy | 7 | Present the full search strategies for all databases, registers, and websites, including any filters and limits used. | Page 02 |
| Selection process | 8 | Specify the methods used to decide whether a study met the inclusion criteria of the review, including how many reviewers screened each record and report retrieved, whether they worked independently, and if applicable, details of automation tools used in the process. | Page 03 |
| Data collection | 9 | Specify the methods used to collect data from reports, including how many reviewers collected data from each report, whether they worked independently, any processes for obtaining or confirming data from study investigators, and if applicable, details of automation tools used in the process. | Page 03 |
| Data items | 10a | List and define all outcomes for which data were sought. Specify whether all results that were compatible with each outcome domain in each study were sought (e.g., for all measures, time points, analyses), and if not, the methods used to decide which results to collect. | Page 02 |
|  | 10b | List and define all other variables for which data were sought (e.g., participant and intervention characteristics, funding sources). Describe any assumptions about missing or unclear information. | Page 02 |
| Risk of bias assessment | 11 | Specify the methods used to assess risk of bias in the included studies, including details of the tool(s) used, how many reviewers assessed each study and whether they worked independently, and if applicable, details of automation tools used in the process. | Page 03 |
| Effect measures | 12 | Specify for each outcome the effect measure(s) (e.g., risk ratio, mean difference) used in the synthesis or presentation of results. | Page 03 |
| **Section and Topic** | **Item #** | **Checklist item** | **Location where item is reported** |
| Synthesis methods | 13a | Describe the processes used to decide which studies were eligible for each synthesis (e.g., tabulating the study intervention characteristics and comparing against the planned groups for each synthesis (item #5)). | Page 03 |
|  | 13b | Describe any methods required to prepare the data for presentation or synthesis, such as handling of missing summary statistics or data conversions. | Page 03 |
|  | 13c | Describe any methods used to tabulate or visually display results of individual studies and syntheses. | Page 03 |
|  | 13d | Describe any methods used to synthesize results and provide a rationale for the choice(s). If meta-analysis was performed, describe the model(s), method(s) to identify the presence and extent of statistical heterogeneity and software package(s) used. | Page 03 |
|  | 13e | Describe any methods used to explore possible causes of heterogeneity among study results (e.g., subgroup analysis, meta-regression). | Page 03 |
|  | 13f | Describe any sensitivity analyses conducted to assess robustness of the synthesized results. | Page 03 |
| Reporting bias assessment | 14 | Describe any methods used to assess risk of bias due to missing results in a synthesis (arising from reporting biases). | Page 03 |
| Certainty assessment | 15 | Describe any methods used to assess certainty (or confidence) in the body of evidence for an outcome. | Page 03 |
| **RESULTS** | | | |
| Study selection | 16a | Describe the results of the search and selection process, from the number of records identified in the search to the number of studies included in the review, ideally using a flow diagram. | Page 03 |
|  | 16b | Cite studies that might appear to meet the inclusion criteria, but which were excluded and explain why they were excluded. | Page 03 |
| Study characteristics | 17 | Cite each included study and present its characteristics. | Page 03-9 |
| Risk of bias in studies | 18 | Present assessments of risk of bias for each included study. | Page 09-10 |
| Results of individual studies | 19 | For all outcomes, present, for each study: (a) summary statistics for each group (where appropriate) and (b) an effect estimates and its precision (e.g., confidence/credible interval), ideally using structured tables or plots. | Page 04-9 |
| Results of syntheses | 20a | For each synthesis, briefly summarise the characteristics and risk of bias among contributing studies. | Page 09-10 |
|  | 20b | Present results of all statistical syntheses conducted. If meta-analysis was performed, present for each the summary estimate and its precision (e.g., confidence/credibility interval) and measures of statistical heterogeneity. If comparing groups, describe the direction of the effect. | Page 10-14 |
|  | 20c | Present results of all investigations of possible causes of heterogeneity among study results. | Page 13-14 |
|  | 20d | Present results of all sensitivity analyses conducted to assess the robustness of the synthesized results. | Page 13-14 |
| **Section and topic** | **Item #** | **Checklist item** | **Location where item is reported** |
| Reporting biases | 21 | Present assessments of risk of bias due to missing results (arising from reporting biases) for each synthesis assessed. | Page 03 |
| Certainty of evidence | 22 | Present assessments of certainty (or confidence) in the body of evidence for each outcome assessed. | Page 13-14 |
| **DISCUSSION** | | | |
| Discussion | 23a | Provide a general interpretation of the results in the context of other evidence. | Page 14-15 |
|  | 23b | Discuss any limitations of the evidence included in the review. | Page 16-17 |
|  | 23c | Discuss any limitations of the review processes used. | Page 16-17 |
|  | 23d | Discuss implications of the results for practice, policy, and future research. | Page 16-17 |
| **OTHER INFORMATION** | | | |
| Registration and protocol | 24a | Provide registration information for the review, including register name and registration number, or state that the review was not registered. | Page 01-2 |
|  | 24b | Indicate where the review protocol can be accessed or that a protocol was not prepared. | Page 02 |
|  | 24c | Describe and explain any amendments to information provided at registration or in the protocol. | NA |
| Support | 25 | Describe sources of financial or non-financial support for and the role of the funders or sponsors in the review. | Page 17 |
| Competing interests | 26 | Declare any competing interests of review authors. | Page 17 |
| Availability of data, code, and other materials | 27 | Report which of the following are publicly available and where they can be found template data collection forms; data extracted from included studies; data used for all analyses; analytic code; any other materials used in the review. | NA |

*From:*  Page MJ, McKenzie JE, Bossuyt PM, Boutron I, Hoffmann TC, Mulrow CD, et al. The PRISMA 2020 statement: an updated guideline for reporting systematic reviews. BMJ 2021;372:n71. doi: 10.1136/bmj.n71

For more information, visit: <http://www.prisma-statement.org/>

**Supplementary Figures Legends**

**Figure S1.** Forest plot for effect of natural compounds/extracts intervention studies assessing ADAS-cog classified by plant structure extract.

*ADAS-cog* Alzheimer Disease Cooperative Study-Activities of Daily Living Scale

**Figure S2.** Forest plot for effect of natural compounds/extracts intervention studies assessing MMSE classified by plant structure extract.

*MMSE* Mini Mental State Examination

**Figure S3.** Funnel plot illustrating the assessment of bias in studies evaluating ADAS-cog.

**Figure S4.** Funnel plot illustrating the assessment of bias in studies evaluating MMSE.

**Figure S5.** Egger plot illustrating the assessment of bias in studies evaluating ADAS-cog.

**Figure S6.** Egger plot illustrating the assessment of bias in studies evaluating MMSE.
