# Supplementary material for: Improving cognitive impairment through chronic consumption of natural compounds/extracts: a systematic review and meta-analysis of randomized controlled trials
Source: Front Aging Neurosci. 2025 Jan 30;16:1531278. doi: 10.3389/fnagi.2024.1531278 (PMC11821934; doi:10.3389/fnagi.2024.1531278)
Supplement: Supplementary file 2 [file Presentation_1.pptx]

## Slide 1
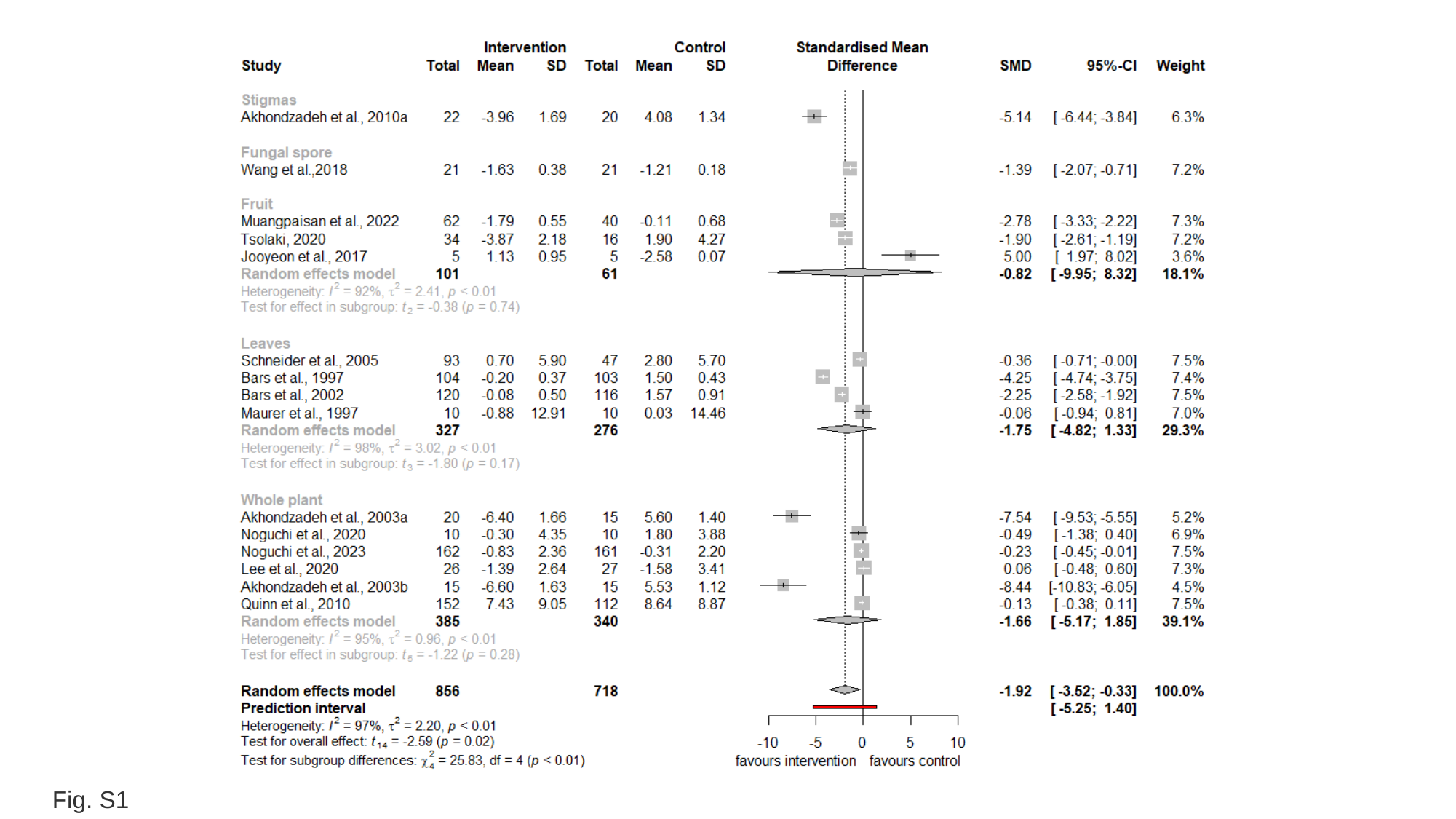

Fig. S1

## Slide 2
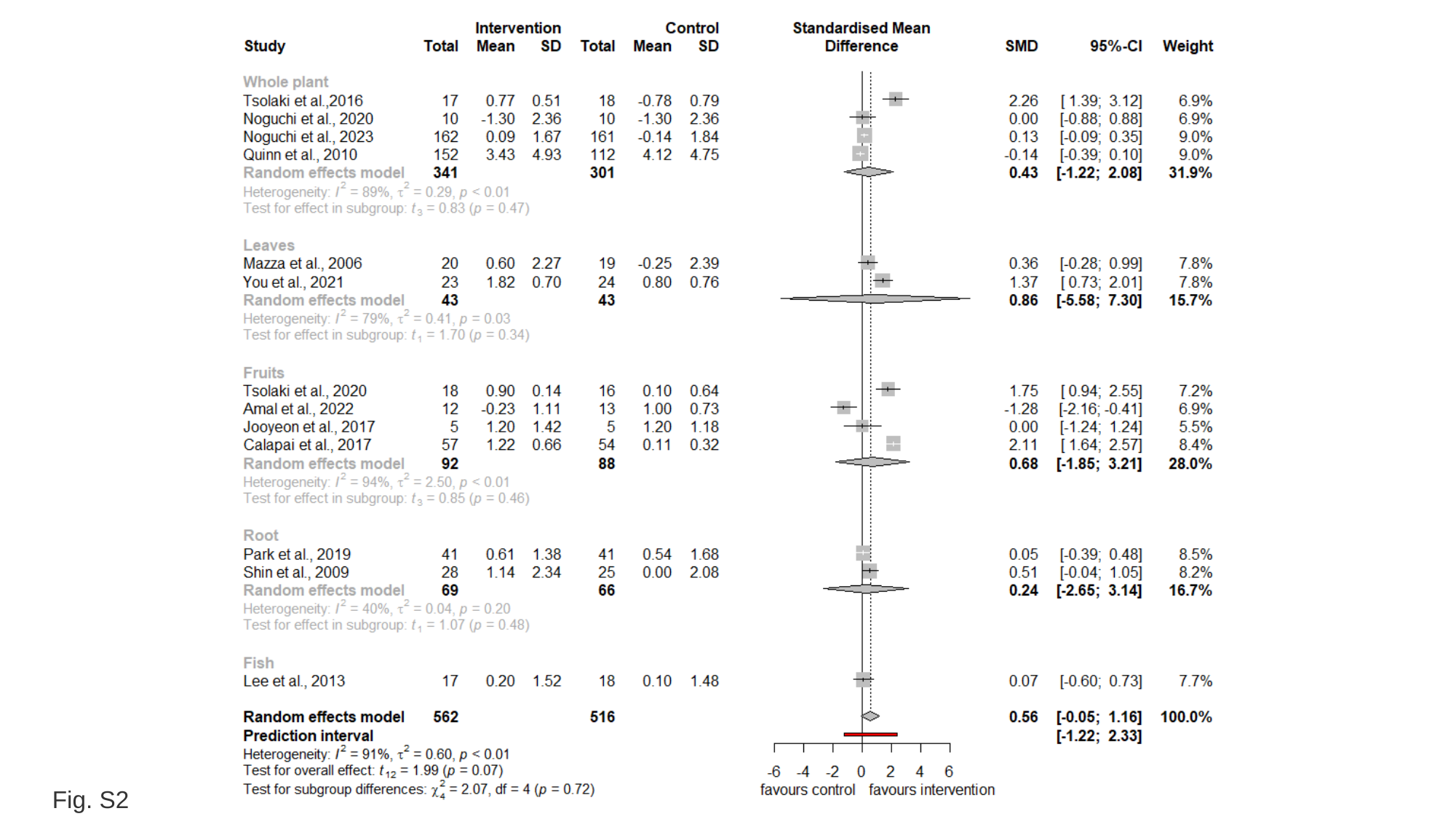

Fig. S2

## Slide 3
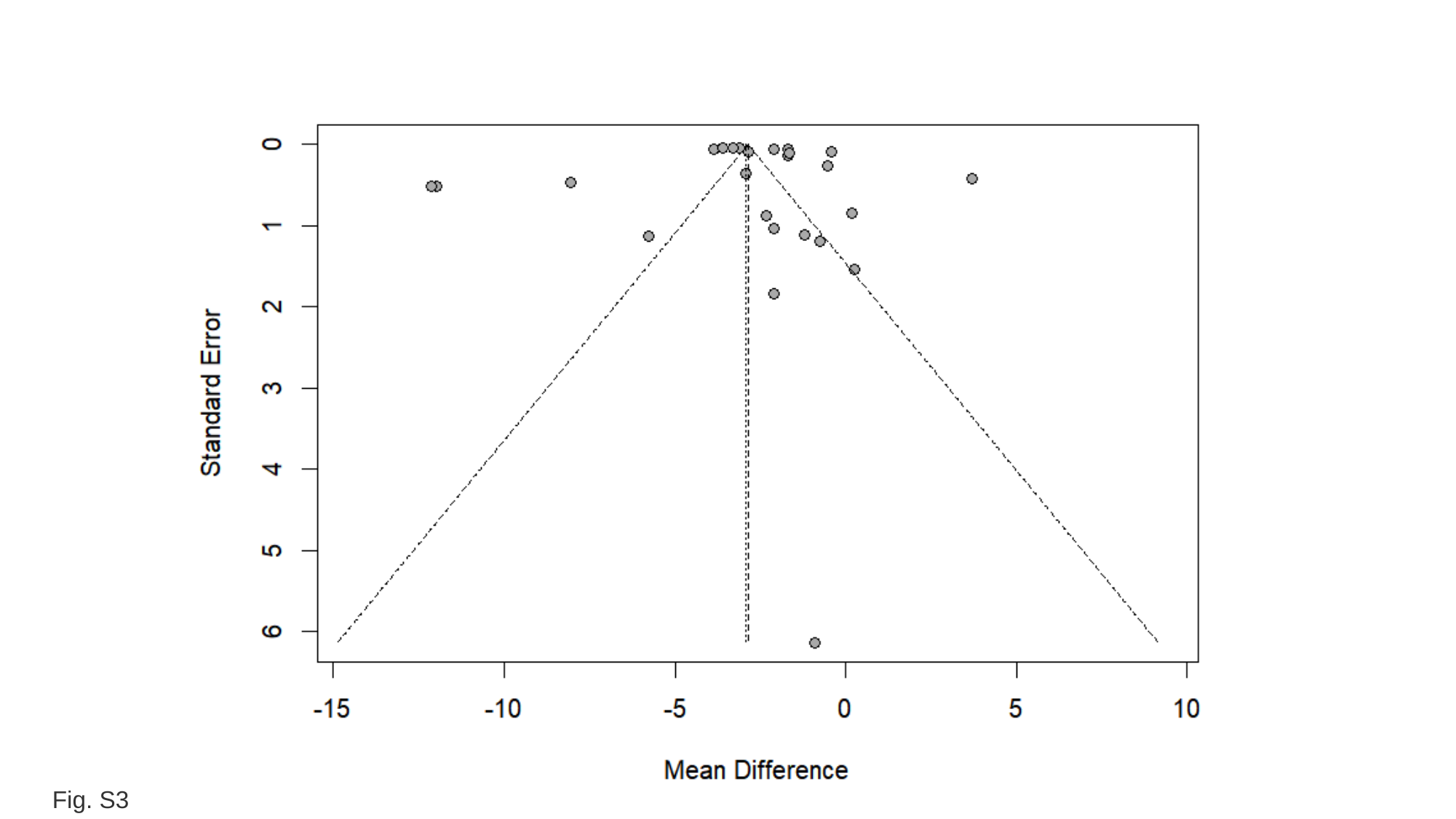

Fig. S3

## Slide 4
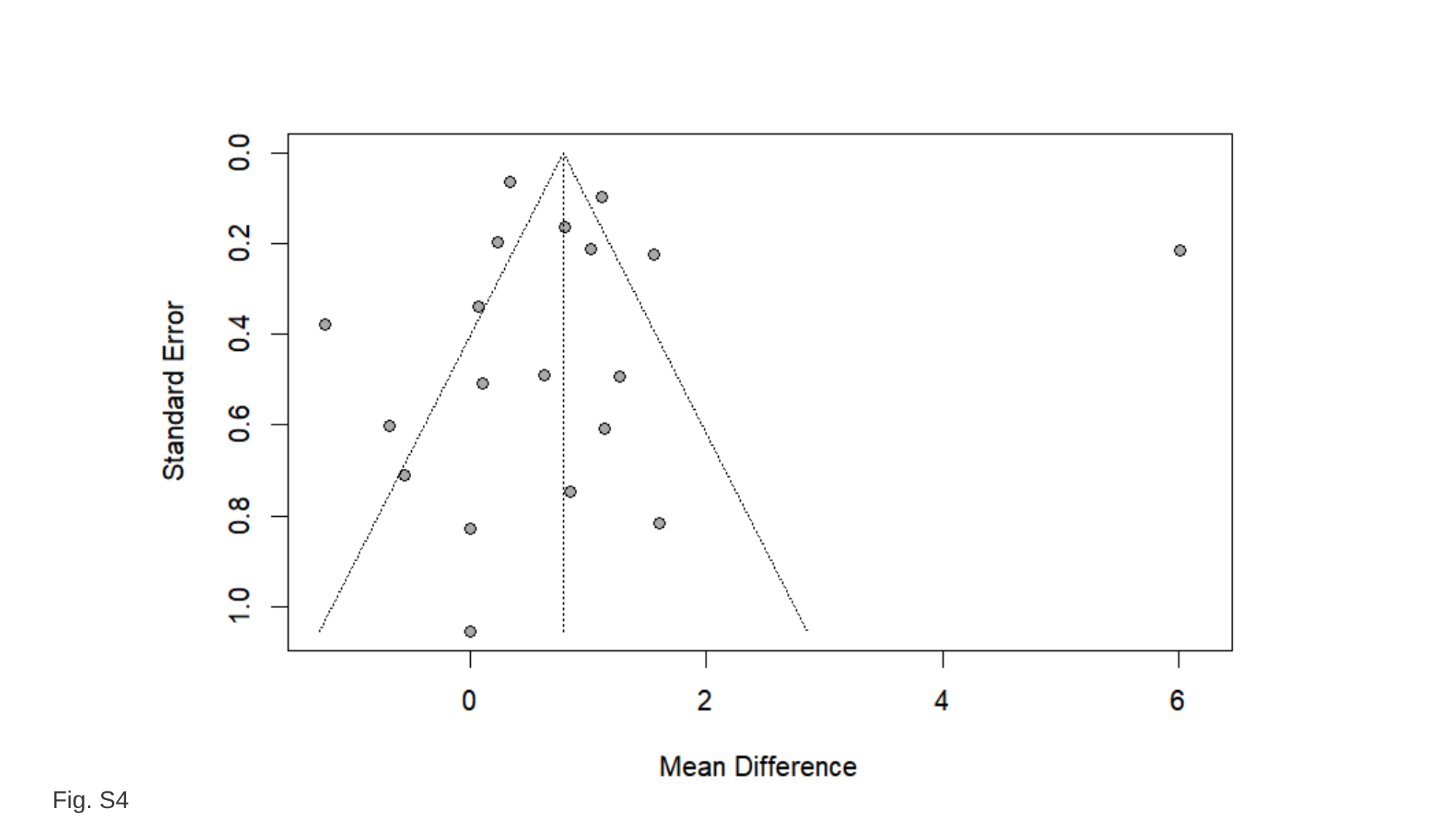

Fig. S4

## Slide 5
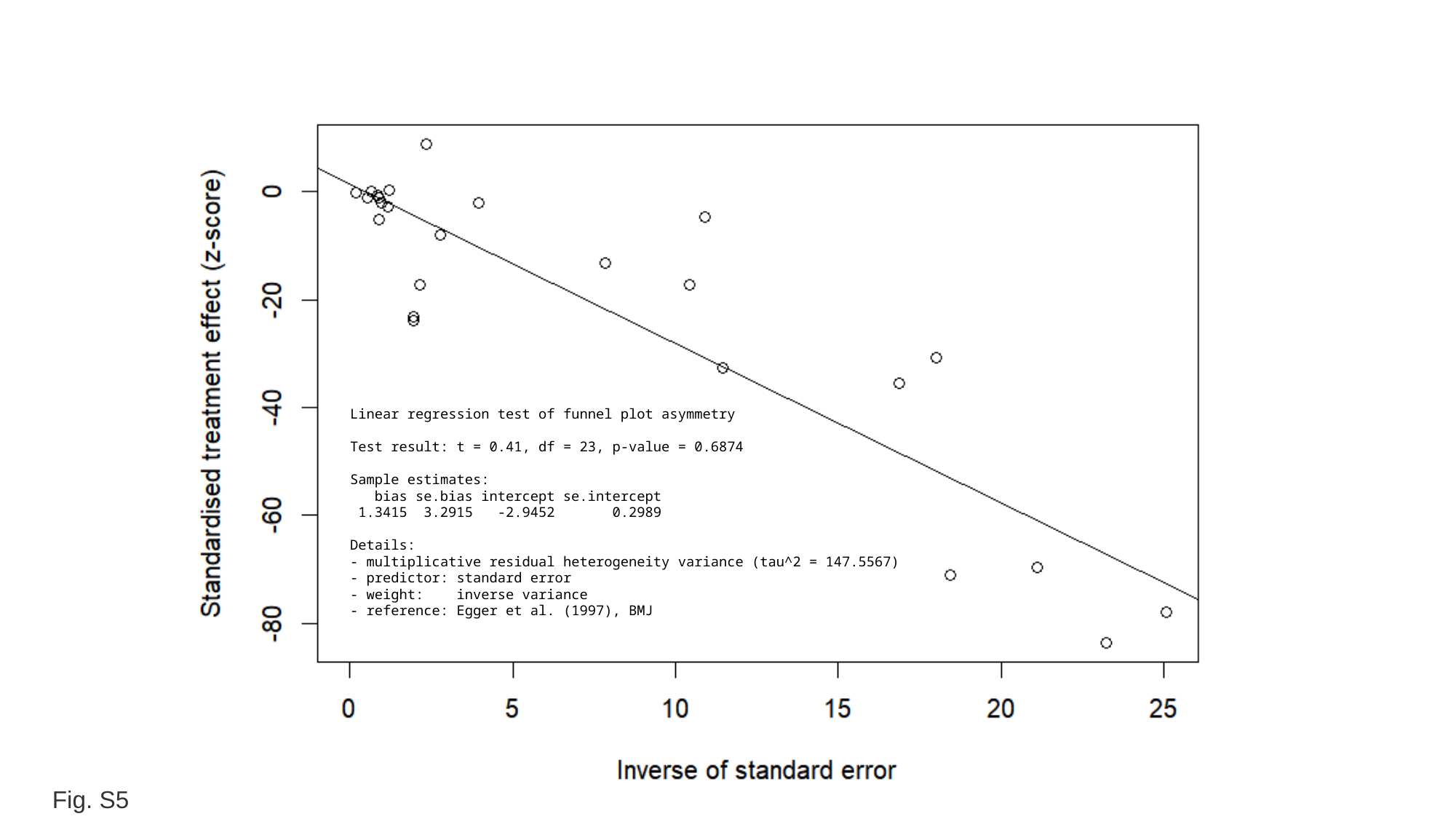

Linear regression test of funnel plot asymmetry
Test result: t = 0.41, df = 23, p-value = 0.6874
Sample estimates:
 bias se.bias intercept se.intercept
 1.3415 3.2915 -2.9452 0.2989
Details:
- multiplicative residual heterogeneity variance (tau^2 = 147.5567)
- predictor: standard error
- weight: inverse variance
- reference: Egger et al. (1997), BMJ
Fig. S5

## Slide 6
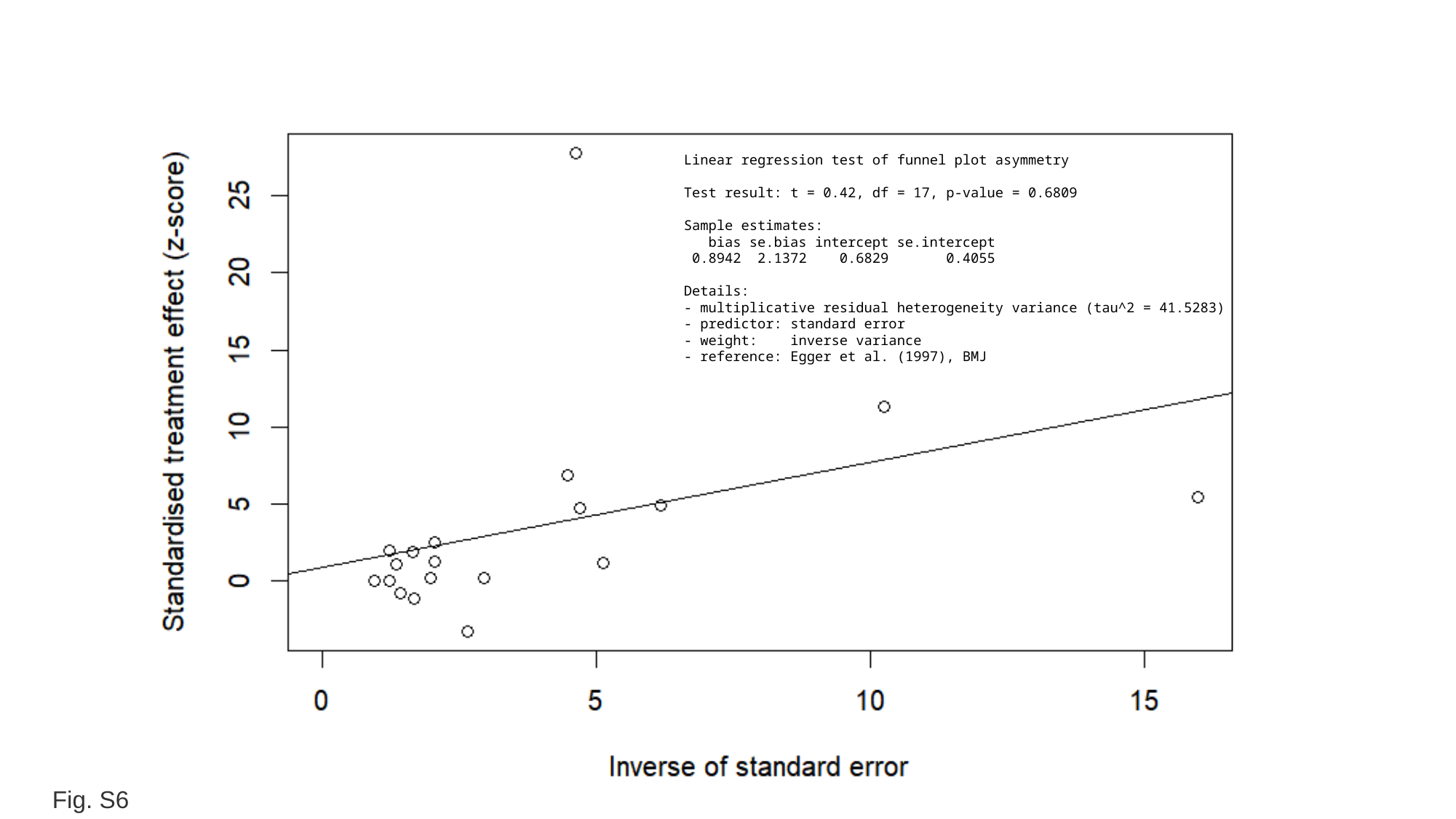

Linear regression test of funnel plot asymmetry
Test result: t = 0.42, df = 17, p-value = 0.6809
Sample estimates:
 bias se.bias intercept se.intercept
 0.8942 2.1372 0.6829 0.4055
Details:
- multiplicative residual heterogeneity variance (tau^2 = 41.5283)
- predictor: standard error
- weight: inverse variance
- reference: Egger et al. (1997), BMJ
Fig. S6
